# A neuron-immune circuit regulates neurodegeneration in the hindbrain and spinal cord of Arf1-ablated mice

**DOI:** 10.1093/nsr/nwad222

**Published:** 2023-08-18

**Authors:** Guohao Wang, Shuhan Jin, Jiaqi Liu, Xu Li, Peng Dai, Yuetong Wang, Steven X Hou

**Affiliations:** The Basic Research Laboratory, Center for Cancer Research, National Cancer Institute at Frederick, National Institutes of Health, Frederick, MD 21702, USA; Department of Cell and Developmental Biology at the School of Life Sciences, State Key Laboratory of Genetic Engineering, Institute of Metabolism and Integrative Biology, Human Phenome Institute, Department of Liver Surgery and Transplantation of Liver Cancer Institute at Zhongshan Hospital, Fudan University, Shanghai200438, China; Department of Cell and Developmental Biology at the School of Life Sciences, State Key Laboratory of Genetic Engineering, Institute of Metabolism and Integrative Biology, Human Phenome Institute, Department of Liver Surgery and Transplantation of Liver Cancer Institute at Zhongshan Hospital, Fudan University, Shanghai200438, China; Department of Cell and Developmental Biology at the School of Life Sciences, State Key Laboratory of Genetic Engineering, Institute of Metabolism and Integrative Biology, Human Phenome Institute, Department of Liver Surgery and Transplantation of Liver Cancer Institute at Zhongshan Hospital, Fudan University, Shanghai200438, China; Department of Cell and Developmental Biology at the School of Life Sciences, State Key Laboratory of Genetic Engineering, Institute of Metabolism and Integrative Biology, Human Phenome Institute, Department of Liver Surgery and Transplantation of Liver Cancer Institute at Zhongshan Hospital, Fudan University, Shanghai200438, China; Department of Cell and Developmental Biology at the School of Life Sciences, State Key Laboratory of Genetic Engineering, Institute of Metabolism and Integrative Biology, Human Phenome Institute, Department of Liver Surgery and Transplantation of Liver Cancer Institute at Zhongshan Hospital, Fudan University, Shanghai200438, China; Department of Cell and Developmental Biology at the School of Life Sciences, State Key Laboratory of Genetic Engineering, Institute of Metabolism and Integrative Biology, Human Phenome Institute, Department of Liver Surgery and Transplantation of Liver Cancer Institute at Zhongshan Hospital, Fudan University, Shanghai200438, China; The Basic Research Laboratory, Center for Cancer Research, National Cancer Institute at Frederick, National Institutes of Health, Frederick, MD 21702, USA

**Keywords:** neurodegenerative diseases, disease-associated microglia, γδ T cells, reactive astrocyte, *IFN-*γ, C3

## Abstract

Neuroimmune connections have been revealed to play a central role in neurodegenerative diseases (NDs). However, the mechanisms that link the central nervous system (CNS) and peripheral immune cells are still mostly unknown. We recently found that specific ablation of the *Arf1* gene in hindbrain and spinal cord neurons promoted NDs through activating the NLRP3 inflammasome in microglia via peroxided lipids and adenosine triphosphate (ATP) releasing. Here, we demonstrate that IL-1β with elevated chemokines in the neuronal Arf1-ablated mouse hindbrain and spinal cord recruited and activated γδ T cells in meninges. The activated γδ T cells then secreted IFN-γ that entered into parenchyma to activate the microglia-A1 astrocyte-C3-neuronal C3aR neurotoxic pathway. Remarkably, the neurodegenerative phenotypes of the neuronal *Arf1*-ablated mice were strongly ameliorated by *IFN-*γ or *C3* knockout. Finally, we show that the Arf1-reduction-induced neuroimmune-*IFN-*γ-gliosis pathway exists in human NDs, particularly in amyotrophic lateral sclerosis and multiple sclerosis. Together, our results uncover a previously unknown mechanism that links the CNS and peripheral immune cells to promote neurodegeneration.

## INTRODUCTION

Neurodegenerative diseases (NDs) are heterogeneous diseases in which multiple detrimental factors contribute to progressive loss of structure and/or function of neuronal cells and brain areas, leading to different and sometimes overlapping symptoms along with functional decline of cognition and/or movement. Although the underlying mechanisms of NDs are not yet well understood, recent genome-wide association studies (GWASs) and sequencing findings suggest a key role of inflammation and neuroimmune communications in different ND diseases [1,2].

In experimental autoimmune encephalomyelitis (EAE) and multiple sclerosis (MS), interactions between central nervous system (CNS)-resident cells and the immune system play a major role in promoting ND [3–5]. They are involved in cross talk among immune cells, neurons, microglia and astrocytes. First, T cell activation induced by a damaged neuron-released myelin autoantigen leads to the production and secretion of proinflammatory cytokines such as TNF, IFN-γ and IL-17. These cytokines exert proinflammatory effects on CNS-resident microglia and astrocytes. Microglia and astrocytes then cross talk through multiple cues, such as the activation of microglia upregulation and the secretion of proinflammatory molecules including IL-1β, IL-1α, TNF and C1q. Astrocytes sense these cues through cell surface receptors. Meanwhile, astrocyte-NK/T cell crosstalk has also been reported [6]. The meningeal NK cells produce IFN-γ to promote CNS inflammation and also induce TRAIL expression in astrocytes, limiting inflammation by inducing apoptosis of T cells. Once activated, pathogenic astrocytes drive myelin and axonal damage through multiple mechanisms, not only by secreting soluble neurotoxic molecules including ROS and NO but also by diminishing metabolic support of neurons as well as reducing neurotrophic factor production. The damaged myelin proteins may further activate T cells to form a vicious cycle.

In other types of NDs, there is compelling evidence that dysfunction in peripheral immunity also contributes to the progression of the diseases [2]. However, the cellular and molecular connections are less clear. Immune cells mostly reside in the brain borders and brain lymphatics or in circulation, but aging and NDs can dramatically increase the T cell population in brains [7]. The activated microglia can also further activate astrocytes to convert a quiescent astrocyte to a reactive astrocyte or disease-associated astrocyte (DAA) [2,8,9]. The pathogenic astrocytes then promote neuronal dysfunction and death through directly neurotoxic molecules, or induce excitotoxicity or reduce neurotrophic factors and nutrients.

Despite compelling indirect evidence, direct connections among peripheral immune cells, neurons, microglia and astrocytes are still lacking in most NDs. In amyotrophic lateral sclerosis (ALS), multiple ALS-related proteins, including TBK1, GRN, C9orf72 and TMEM106B, are implicated in endosomal sorting and endosomal lysosomal function [10–12]. Abnormal protein and lipid sorting and transport may be intrinsic to the lesion. Neuroimmune abnormalities are also an important cause of ALS/frontotemporal dementia (FTD) [2,13,14]. However, the connection between neuroimmune abnormalities and abnormal protein/lipid sorting/transportation in ALS lesions is also still unclear.

The ADP-ribosylation factor 1 (Arf1) is a Ras superfamily GTPase that plays an important molecular switching role in regulating various biological processes such as intracellular protein and lipid sorting and transport [15–17]. GTPase activating proteins (GAPs) and guanine exchange factors (GEFs) catalyze the conversion between GTP- to GDP- and GDP- to GTP-bound enzymes, respectively. Site-specific activation and inactivation of Arf1 may be done by a GEF or GAP localized by different organelles. Further, both gain- and loss-function of *C9orf72* are associated with LS-FTD disease. It was recently found that C9orf72 has Arf1 GAP function [18,19].

We previously found that the Arf1-ACSL (acyl-CoA synthetase long chain)-mediated lipid metabolism sustains stem cells and cancer stem cells in *Drosophila* and mice. Its ablation resulted in stem cell necrosis and also triggered an antitumor immune response [20,21]. We recently found that the neuronal Arf1 ablation resulted in accumulation of lipid droplets as well as production and transfer of peroxidized lipids that activated the NLRP3 inflammasome in microglia, which facilitated the release of IL-1β and promoted neurodegeneration through the neurotoxic reactive astrocytes. In humans, low Arf1 protein levels and accompanying microglia-astrocyte activation are closely associated with NDs, particularly ALS and MS [22]. *ACSL5* was recently discovered as a novel risk gene associated with sporadic ALS in a large multi-ethnic meta-analysis among Japanese, European and Chinese populations [23], suggesting that the Arf1-ACSL-regulated lipid metabolism may play an essential role in ALS pathophysiology. Together, these data suggest that elucidating Arf1-mediated neuronal biological function could be a breakthrough in solving the mystery of ALS-FTD.

In this study, we found that in the neuronal Arf1-ablated mouse brain, IL-1β together with elevated chemokines recruited and activated γδ T cells in meninges. The activated γδ T cells then secreted IFN-γ, which entered into parenchyma to activate the microglia-A1 astrocyte-C3 neurotoxic pathway, which leads to the destruction of neurons. We demonstrated that the neurodegenerative phenotypes of the *Arf1*-ablated mice were strongly ameliorated by knockout of *IFN-γ, Rag1* and C3 but not of *TLR4*. The mutant phenotypes were also significantly rescued by neutralizing antibodies of VLA-4, the γδ T cell receptor and IFN-γ. Finally, we show that the Arf1-reduction-induced neuroimmune-IFN-*γ*-gliosis pathway exists in human NDs, particularly in ALS and MS. Our results together uncovered a previously unknown circuit that links neuron, microglia, peripheral immune cells, astrocyte and neuron to promote neurodegeneration.

## RESULTS

### Arf1 ablation promotes neurodegeneration through the IFN-**γ**/reactive astrocyte pathway

**Figure 1.**
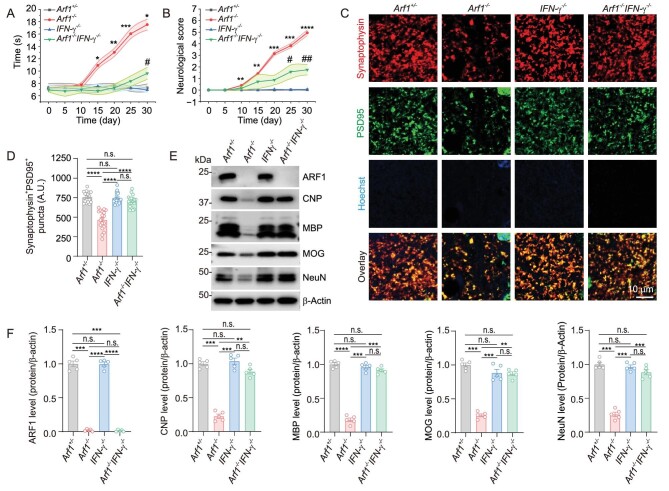
Arf1 ablation promotes neurodegeneration through IFN-γ. (A) Balance beam test and (B) neurological score of mice with the indicated genotypes (*n* = 5 per group, data from three independent experiments). (C and D) Immunofluorescence staining for PSD95 and synaptophysin (C) and quantification (D) of PSD95- and synaptophysin-immunoreactive dots in spinal cord sections of mice with the indicated genotypes, after injection with tamoxifen on day 22 (*n* = 19 per group from 5 mice). (E) Western blotting with indicated antibodies of the spinal cord lysates from mice with indicated genotypes, after injection of tamoxifen 22 days. Scale bar: 10 μm. (F) Quantification of (E) (*n* = 5 per group). Data are represented as mean ± SEM. *P < 0.05, **P < 0.01, ***P < 0.001, ****P < 0.0001 using two-way ANOVA (A and B) and one-way ANOVA (D and F), with Bonferroni multiple comparison test.

**Figure 2.**
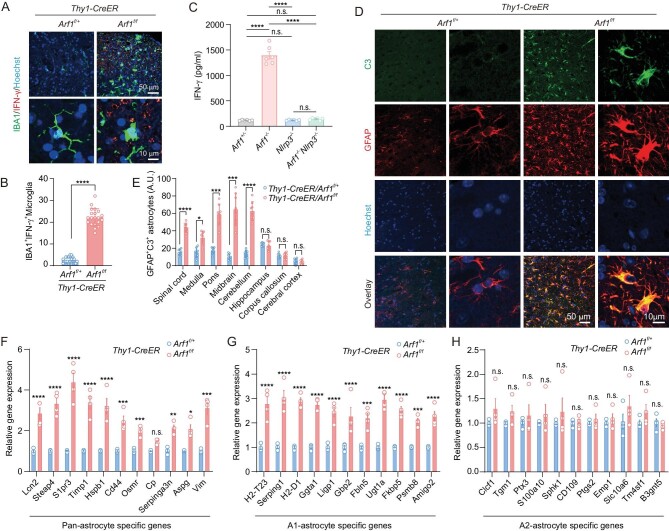
Arf1 ablation promotes neurodegeneration via an IFN-γ-reactive astrocyte-C3 pathway. (A) IFN-γ protein level and IBA1-immunoreactive microglia were significantly increased in *Arf1*-ablated mice. Scale bar: 50 μm (top), 10 μm (bottom). (B) Quantification of IFN-γ- and IBA1-immunoreactive microglia in the spinal cords of Arf1-ablated and control mice. *n* = 20 sections from 5 mice in each group, ****P < 0.0001 using unpaired t-test. (C) Quantification of IFN-γ in the spinal cords of control, *Arf1*-ablated and *Arf1*-ablated-with-*Nlrp3*-deficiency mice. *n* = 6 mice per group, n.s. means no significant difference, *P < 0.05, **P < 0.01, ***P < 0.001, ****P < 0.0001 using one-way ANOVA with Bonferroni multiple comparison test. (D) Immunofluorescence staining for GFAP and C3 in spinal cords from mice with the indicated genotypes. Scale bars: 50 μm and 10 μm. (E) Quantification of GFAP^+^ C3^+^ astrocytes in different brain areas of mice with the indicated genotypes (*n* = 8 per group). Note the increased GFAP/C3-positive astrocytes of Arf1-ablated mice in areas of the hindbrain and midbrain but not the forebrain. n.s. means no significant difference, *P < 0.05, **P < 0.01, ***P < 0.001, ****P < 0.0001 using two-way ANOVA with Bonferroni multiple comparison test. (F–H) Relative gene expression of the (F) pan-astrocytes, (G) A1 astrocytes and (H) A2 astrocytes was assayed in the spinal cord of control and Arf1-ablated mice by qPCR analysis (*n* = 4 per group). The experiment was conducted on the 22nd day after mice were injected with tamoxifen. Data are represented as mean ± SEM. n.s. means no significant difference, *P < 0.05, **P < 0.01, ***P < 0.001, ****P < 0.0001 using two-way ANOVA with Bonferroni multiple comparison test.

The ablation of Arf1 in neurons through a focused approach resulted in a pronounced phenotype of neurological disorder (Supplementary Table 1). As mentioned above, we recently found that peroxidized lipids released from the Arf1-ablated neurons activated a NLRP3 inflammasome-IL-1β pathway in microglia and also induced the neurotoxic reactive astrocytes [22]. However, it is not clear how the IL-1β connects to astrocyte activation. In mouse tumors, we previously found that Arf1 ablation in cancer stem cells promoted an IFN-γ-mediated antitumor immune response [21]. To assess the potential role of IFN-γ in mediating the neurodegenerative pathway in the *Arf1*-ablated mice (*Thy-1-CreER/Arf1*^f/f^, *Arf1*^−/−^), we examined the phenotypes of the *Arf1*-ablated mice in an *IFN-γ*-deficient background (*Arf1*^−/−^*IFN-γ*^−/−^) (Fig. 1). We found that the slow traveling in the balance beam tests, the poor neurological score, synapse loss, axon demyelination and axon degeneration associated with Arf1-ablated mice were almost completely suppressed in IFN-γ-deficient mice (Fig. 1A–F and Fig. S1A–F). The IFN-γ protein level and activated microglia were significantly increased (Fig. 2A, B, Figs S1G and S2A–C) in the *Arf1*-ablated mice but the increase was dramatically suppressed in *Nlrp3-*deficient mice (Fig. 2C), indicating that the increase might occur downstream of NLRP3 inflammasome activation. Further, the increased activated microglia were not colocalized with the lysosomal protein CD68 (Fig. S2D), suggesting that they were not activated to engulf synaptic material as previously described in Alzheimer mouse models [24]. Furthermore, reactive A1 astrocytes and complement C3 were significantly increased (Fig. 2D–H, Fig. S2A–C) in the Arf1-ablated mice, but their increase—as well as the increase of activated microglia—were dramatically suppressed in *Arf1* and *IFN-γ* double-knockout mice (Fig. S3A–D). However, the increase in activated microglia, A1 astrocytes and C3, as well as their suppression by IFN-γ deficiency, was restricted to the hindbrain, midbrain and spinal cord, not the forebrain (Fig. 2E, Figs S2B, C and S3B–E). The levels of IFN-γ, TNF, IL-1α and C3, but not C1q, were also significantly elevated in Arf1-deficient mice but suppressed in *Arf1* and *IFN-γ* double-knockout mice (Fig. S4A–I). Our previous work demonstrated that TNF and IL-1β were selectively induced in microglia and then regulated C3 expression in astrocytes [22].

These data collectively suggest that IFN-γ is a major downstream component in the Arf1-ablation-induced neurodegenerative pathway, functions downstream of the peroxidized-lipids–microglial-NLRP3-inflammasome-IL-1β pathway [22], and possibly activates the A1-astrocytes–C3 pathway to damage neurons and oligodendrocytes [9,25].

### IFN-**γ** mainly comes from meningeal **γδ** T cells

**Figure 3.**
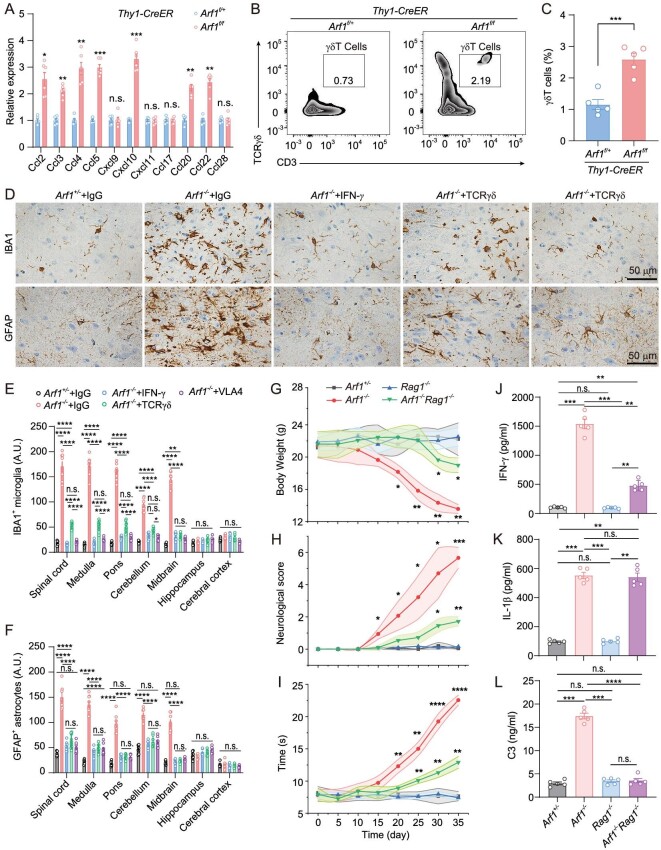
IFN-γ mainly comes from meningeal γδ T cells. (A) Expression of chemokines in spinal cords of control and Arf1-ablated mice. *n* = 6 mice per group, n.s. means no significant difference, *P < 0.05, **P < 0.01, ***P < 0.001, ****P < 0.0001 using two-way ANOVA with Bonferroni multiple comparison test. (B and C) Meninges were dissected from control and Arf1-ablated mice; single-cell suspensions were immunostained (B). T cells were gated on live, single, CD3^+^, TCRγδ^+^ events and counted by flow cytometry (*n* = 5 mice per group) and quantification (C). n.s. means no significant difference, **P < 0.01, using unpaired t-test. (D–F) Injection of antibodies of IFN-γ, VLA-4 and γδ T cell receptor significantly suppressed the phenotypes of Arf1-ablated mice, including induction of (D and E) IBA1^+^ microglia and (D and F) GFAP^+^ astrocytes. *n* = 8 per genotype. Control (*Thy-1-CreER/Arf1^f/+^, Arf1^+^^/^^−^*), *Rag1*-deficient (*Rag1*^−/−^), *Arf1*-ablated (*Thy-1-CreER/Arf1^f/f^, Arf1*^−/−^) and *Arf1*-ablated*-*in-a-*Rag1*-deficient-background *(Thy-1-CreER/Arf1^f/f^/Rag1*^−/−^*, Arf1^−^^/^^−^Rag1*^−/−^) mice were assayed on the following indicated phenotypes. (G–I) Body weight (G), neurological score (H) and balance beam test (I) of mice with indicated genotypes (*n* = 5 mice per group). (J–L) Quantification of (J) IFN-γ, (K) IL-1β and (L) C3 in the spinal cord lysates of mice with the indicated genotypes (*n* = 5 mice per group). Data are represented as mean ± SEM. *P < 0.05, **P < 0.01, ***P < 0.001 using two-way ANOVA (E–I) and one-way ANOVA (J–L) with Bonferroni multiple comparison test.

We further assessed expression of chemokine genes and found that several chemokines were significantly increased in the Arf1-ablated mice, including CCL2, CCL3, CCL4, CCL5, CCL20, CCL22 and CXCL10 (Fig. 3A). Furthermore, our cell culture experiments suggested that CCL2, CCL5 and CCL22 were from neuronal cells, CCL4 and CXCL10 were from microglia, and CXCL10 and CCL20 were from astrocytes (Fig. S5A–C). CXCL10 and CCL5 chemokines were reported to stimulate CD4^+^ and CD8^+^ lymphocyte migration into the intra-tumor and stromal compartment [26], and CCL20 drove brain infiltration of Treg cells [27]. They may have similar roles in recruiting T cells into the brains of Arf1-ablated mice.

T cells are the major source of IFN-γ. We examined immune cells isolated from brain parenchyma by fluorescence-activated cell sorting analysis (Fig. S6A–C). We found that T and B cells did not change, and only macrophages (CD11b^hi^F4/80^hi^) and monocytes (CD11b^hi^Ly6G/C^hi^) were significantly increased in Arf1-ablated mice. CD11b and F4/80 are also microglial markers, and Ly6G/C also labels microglial precursors [28]. These changes might actually reflect the increased microglia as described in the earlier section.

We next used fluorescence-activated cell sorting analysis to examine immune cells isolated from brain meninges (Fig. 3B, C, Fig. S7) and found that only γδ T cells were significantly increased. We then injected antibodies of IFN-γ, VLA-4 (an integrin required for T cell CNS homing [29]) and γδ T cell receptor into the Arf1-ablated mice. Partial elimination of IFN-γ and γδ T cells significantly suppressed the phenotypes of the Arf1-ablated mice (Fig. 3D–F). These data collectively suggest that the meningeal γδ T cells are the one resource of IFN-γ that drives neurodegeneration in the Arf1-ablated mice. Consistent with our finding, a recent publication found that γδ T cells in cerebrospinal fluid provide the early source of IFN-γ that aggravates lesions in spinal cord injuries [30].

We further generated the Arf1 and Rag1 double-knockout mouse (*Arf1^−^^/^^−^Rag1^−^^/^^−^*) (Fig. 3G–L) and found that Rag1 knockout significantly suppressed the phenotypes of the Arf1-ablated mice (Fig. 3G–I, Fig. S8A–J). Furthermore, Rag1 knockout significantly blocked induction of IFN-γ and C3, but not IL-1β, in the Arf1-ablated mice (Fig. 3J–L). These data collectively suggested that the meningeal γδ T cells recruited by activated microglia and the Arf1-ablated neuron might be the major resource of IFN-γ that drove neurodegeneration in the Arf1-ablated mice.

### IFN-**γ** stimulates a subpopulation of microglia with DAM signatures

**Figure 4.**
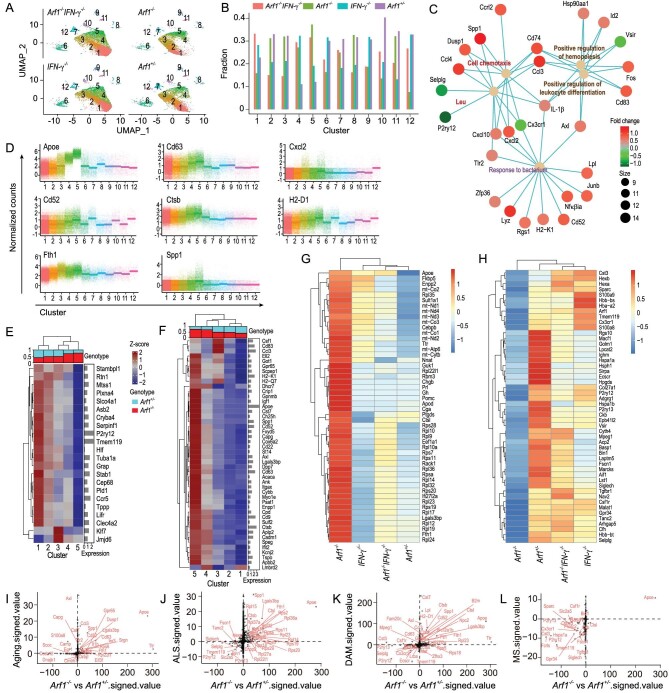
IFN-γ stimulates a subpopulation of microglia with DAM signatures. (A) Uniform manifold approximation and projection map of different clusters from single-microglia cell sequencing results in control, Arf1^−/−^, IFN-γ ^−/−^ and Arf1^−/−^IFN-γ ^−/−^mice. *n* = 5 mice pool together per group for sorting single cells. (B) Cell ratios from clusters 1 to 12 in the four genotypes of microglia. (C) Network analysis of cluster 5 vs. cluster 1 in the microglia from *Arf1^−^^/^^−^* mice. (D) Representative top eight high-expression genes in cluster 5 compared with other clusters. (E and F) Heat map showing cluster 1 to cluster 5 (E) down- and (F) upregulated genes compared with disease-associated microglia (DAM) genes in ALS and DAM. (G and H) Heat map showing the top 50 (G) upregulated and (H) downregulated genes in the Arf1^−/−^ group compared to the other three groups. (I–L) Expression plot comparing *Arf1^−^^/^^−^* vs. control (*Arf1^+/^^−^*) single-cell RNA sequence data with published RNA-sequencing data of microglia in (I) aging, (J) ALS, (K) DAM and (L) multiple sclerosis.

To investigate the underlying state changes of microglia induced by Arf1-ablated neuron and γδT cells, we isolated microglia and used scRNA-seq-analyzed transcriptomes from control (Thy-1-CreER/Arf1^f/+^), Arf1^−/−^ (Thy-1-CreER/Arf1^f/f^), IFN-γ-knockout (IFN-γ^−/−^) and Arf1^−/−^IFN-γ^−/−^ (Thy-1-CreER/Arf1^f/f^; IFN-γ^−/−^) mice. We performed uniform manifold approximation and projection (UMAP) clustering and found a subpopulation of microglia (cluster 5) significantly increased in the Arf1-ablated mouse and mostly restored by IFN-γ knockout (Fig. 4A, B, G–H). Cluster 1 to cluster 5 microglia were identified as homeostatic (cluster 1) to Arf1-ablated-associated microglia (cluster 5). To determine the transcriptome changes, we also compared cluster 5 with cluster 1 in Arf1^−/−^ mice and found that cluster 5 was mainly relative to leukocyte chemotaxis and migration, tumor necrosis factor production and regulation of the inflammatory response pathway (Fig. 4C). Cluster 5 high expression genes include *Apoe, Cd63, Cxcl2, Cd52, Ctsb, H2-D1, Fth1* and *Spp1* (Fig. 4D), and cluster 5 has 22 downregulated and 46 upregulated genes that are similar to disease-associated microglia (DAM) and ALS (Fig. 4E and F). All the up- and down-regulated genes were also identified in Arf1^−/−^ microglia compared with the other three groups (Fig. 4G and H). We further compared the transcriptional profiles of Arf1-ablation-associated microglia with those of microglia in old age [31] (Fig. 4I), ALS [32] (Fig. 4J), DAM [33] (Fig. 4K) and MS [34] (Fig. 4L), and found significant overlap of genes in the microglia of Arf1-ablated mice and the microglia in these disease models. The overlap includes reduced expression levels of several microglia homeostatic genes, including *P2ry12, P2ry13, Tmem119, Selplg, Cx3cr1, Hexb* and *Siglech*, as well as upregulation of six genes: *Spp1, Ctsb, Fth1, Cd63, CD52* and *Apoe*. Among these six, *Apoe* was one of the most upregulated. *Apoe* upregulation in microglia is a major molecular signature of NDs, and the APOE pathway plays an important role in microglial changes in NDs [34]. In addition, the genes that are either downregulated or upregulated in Arf1-ablated microglia are more similar to genes in stage 1 DAM, suggesting that the microglia in Arf1-ablated mice were activated by a Trem2-independent pathway [33].

### IFN-**γ** activates the STAT1 pathway in microglia

We also generated *Arf1* deletion in *Tlr4*-deficient mice and found that *Tlr4* deficiency did not affect the neurodegenerative phenotypes (Fig. S9A), suggesting that the neurotoxic reactive astrocytes in this situation were not induced by activated microglia through the TLR4–NF-κB pathway as previously reported [9,25]. Microglia are known to express the IFN-γ receptor (IFNR), which activates the STAT1-mediated signal transduction pathway to induce expression of TNF and IL-1α [29,35]. We found that STAT1 phosphorylation was significantly increased in the spinal cords, medullas and cerebellums of Arf1-deficient mice (Fig. S9B). Double ablation Arf1 and IFN-γ could restore the STAT1 phosphorylation level (Fig. S9C). In the cultured microglia, IFN-γ treatment significantly activated microglia with enhanced STAT1 phosphorylation as well as increased expression of TNF and IL1α (Fig. S9D and E). As described in Fig. 4, these microglia express signatures of classic DAM and exist in *Arf1^−^^/^^−^* but not in *Arf1^−^^/^^−^IFN-γ^−^^/^^−^* mice. This information together suggests that IFN-γ likely first activated microglia through the IFN-γ-IFNR–STAT1 pathway to produce TNF and IL1α that further activated astrocytes that became the neurotoxic reactive astrocytes in the Arf1-ablated mice.

### C3 promotes axon demyelination in Arf1-ablated mice

**Figure 5.**
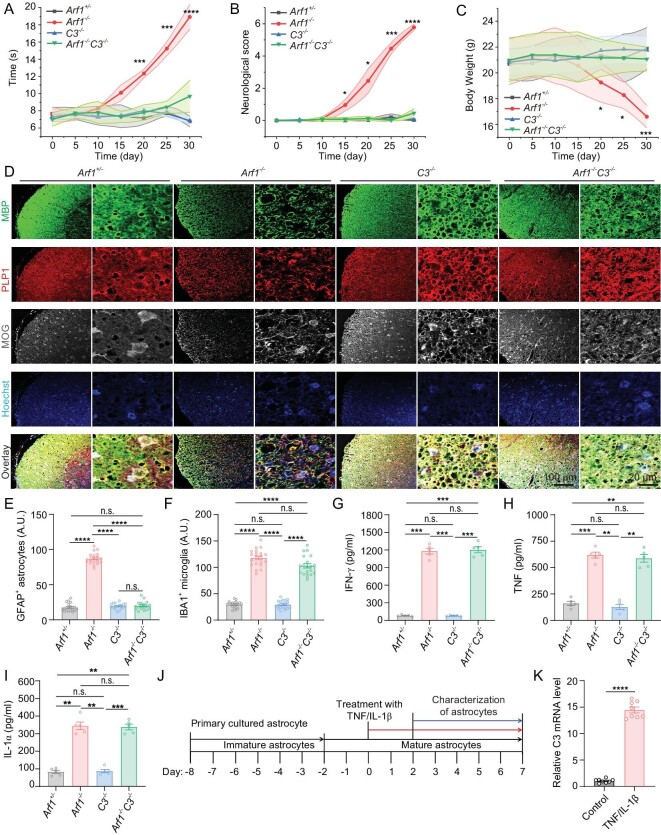
C3 knockout inhibits the neurodegenerative phenotypes of Arf1-ablated mice. (A–C) *C3* deficiency almost completely suppressed major neurodegenerative phenotypes of Arf1-ablated mice, including in (A) balance beam test, (B) neurological score and (C) body weight of mice with indicated genotypes (*n* = 5 per group). Data are represented as mean ± SEM. *P < 0.05, **P < 0.01, ***P < 0.001 using two-way ANOVA with Bonferroni multiple comparison test. (D) Immunofluorescence staining of oligodendrocytes marker, MBP, PLP1 and MOG in indicated mice spinal cord. Nuclear was stained with Hoechst. (E–I) Quantification of (E) GFAP-positive astrocytes, (F) IBA1-positive microglia, (G) IFN-γ, (H) TNF and (I) IL-1α in the spinal cord lysates of mice with the indicated genotypes (*n* = 5 mice per group, (E): *n* = 17 images per group from 5 mice, (F): *n* = 19 images per group from 5 mice). Data are represented as mean ± SEM. *P < 0.05, **P < 0.01, ***P < 0.001 using one-way ANOVA with Bonferroni multiple comparison test. (J) Experimental set-up for activation of astrocytes from primary astrocyte. (K) The level of C3 was measured by quantitative real-time-PCR from activated astrocytes conditional medium in control, TNF and IL-1β treatment (*n* = 8 per group). Data are represented as mean ± SEM. ***P < 0.001, **** *P* < 0.0001 using unpaired t-test.

We further knocked out *Arf1* in neurons in *C3*-deficient mice and found that *C3* deficiency significantly suppressed the major neurodegenerative phenotypes of the Arf1-ablated mice (Fig. 5), including slow traveling in the balance beam tests (Fig. 5A), poor neurological score (Fig. 5B), body weight loss (Fig. 5C), astrocyte activation (Fig. 5E, Fig. S10A and B) and reduction of myelination-related proteins (Fig. 5D, Fig. S10C and D). However, *C3* deficiency did not suppress some neurodegenerative phenotypes of the Arf1-ablated mice, including activated microglia (Fig. 5F, Fig. S10A) and induction of IFN-γ (Fig. 5G), TNF (Fig. 5H) and IL-1α (Fig. 5I). We also treated astrocytes with TNF and IL-1β, and found that the expression level of C3 was robustly increased by TNF and IL-1β treatment (Fig. 5J and K). These data together suggest that C3 may function downstream of active microglia, IFN-γ, TNF and IL-1β, but upstream of reactive astrocytes.

**Figure 6.**
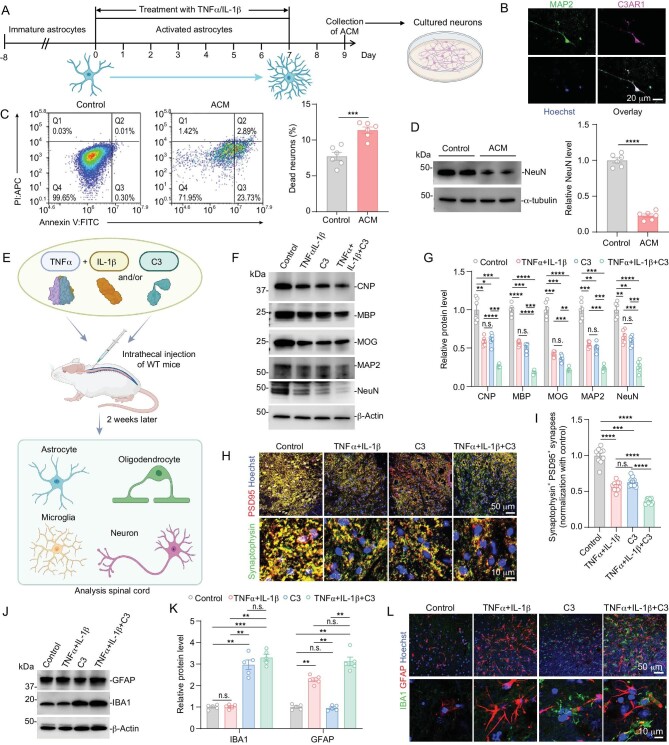
Reactive-astrocyte-damaged-neuron through a C3-C3aR1 pathway. (A) Experimental set-up for collecting ACM (astrocyte conditional medium) from activated primary astrocytes and using ACM to treat primary cultured neurons. The astrocytes were stimulated for 7 days and then changed the new medium of cultured astrocytes. The medium was collected after 48 h, and 500 μL of it was added into 500 μL neuron medium and the medium was mixed by pipette, up and down several times. The mixed medium was added into primary cultured neurons. The total number of surviving and dead neurons was analyzed by P1/Annexin V staining after 48 h. (B) Immunofluorescence staining of MAP2, C3aR1 and Hoechst in primary cultured neurons. (C) Representative plots of the flow cytometry analysis of primary neurons, carried out to detect neuron death (*n* = 6 per group). Quantitation of the ratios of dead primary neurons on flow cytometry. ****P < 0.0001 using two-way ANOVA with Bonferroni multiple comparison test. (D) Western blot analysis of NeuN in control and ACM-treated primary cultured neurons. α-tubulin served as a loading control (*n* = 6 per group), ****P < 0.0001 using two-way ANOVA with Bonferroni multiple comparison test. (E) Experiment set-up for treated C57BL/6J mice with TNF + IL-1β and/or C3 proteins. (F and G) Immunoblot (F) and quantification (G) of oligodendrocytes markers (CNPase, MBP, MOG) and neuron marker (MAP2 and NeuN) in the spinal cord from control, TNF + IL-1β, C3 and TNF + IL-1β +C3 treated mice (*n* = 6 per group). β-Actin was used as loading control. Data are represented as mean ± SEM. *P < 0.05, **P < 0.01 using two-way ANOVA with Bonferroni multiple comparison test. (H and I) Immunofluorescence (IF) staining (H) and quantification (I) of mice spinal cords that were treated with indicated cytokines (*n* = 10 per group). Data are represented as mean ± SEM. *P < 0.05, **P < 0.01, ***P < 0.001, ****P < 0.0001 using one-way ANOVA with Bonferroni multiple comparison test. (J) Western blot of mice spinal cords that were treated with indicated proteins. (K) Quantification of (J) (*n* = 5 mice per group). Data are represented as mean ± SEM. *P < 0.05, **P < 0.01, ***P < 0.001, ****P < 0.0001 using two-way ANOVA with Bonferroni multiple comparison test. (L) IF staining of microglia (IBA1) and astrocytes (GFAP) in the indicated mice that had treatment with PBS (Control), TNF + IL-1β, C3 and TNF + IL-1β+C3 proteins.

We further studied the neuronal toxicity of astrocyte-secreted C3. We cultured N2A cells with astrocyte conditional medium (ACM) collected from TNF- and IL-1β-treated astrocytes. TNF- and IL-1β-treated astrocytes increased the C3 expression level (Fig. S10E and F). To mimic physiological conditions, we repeated this experiment with cultured primary neurons and astrocytes, and found that ACM also promoted neuron death (Fig. 6A–D). We also characterized the C3 receptors in mouse spinal cords and primary cultured neurons and microglia. We found that the C3aR was expressed in the neurons and microglia, the C3aR1 was expressed in the neurons and the CD11b mainly existed in the microglia (Fig. 6B, Fig. S11A–D). Next, we added purified C3 protein to a culture medium of neurons, which also induced the death of cultured primary neurons (Fig. S11E and F). Furthermore, we found that the C3a receptor (C3aR1) was expressed on the neuron surface and C3aR1 knockdown in neurons could rescue neuron death induced by an ACM (Fig. S11G and H). Eliminating C3 by adding C3 antibodies could reduce neuron death induced by an ACM (Fig. S11I). Long-chain saturated lipids were reported to participate in neuron toxicity [36]. We removed lipids from the ACM by passing the ACM through a de-fat column and found that fatty-acid-free ACM could still effectively induce neuron death (Fig. S11J), suggesting that C3 rather than long-chain saturated lipids was a main neurotoxic factor in our system.

We also carried out an intrathecal injection of TNF, IL-1β and/or C3 to the spinal cord of wild type mice and found that both TNF + IL-1β, and C3, could induce demyelination, synapse loss and neuronal death (Fig. 6E–I). A single injection of TNF and IL-1β can induce the activation of astrocytes, and a single injection of C3 can partially activate the microglia (Fig. 6J–L). These results demonstrated that the C3-C3aR1 pathway mediated the neurotoxic function of activated A1 astrocytes in the Arf1-ablated mice. These data suggested that activated astrocytes promoted neuron death.

### The Arf1-ablation-induced IFN**γ**-DAM-A1 astrocyte-C3 pathway exists in MS and ALS patients

In a recent publication [22], we investigated the Arf1-downregulation-induced neurodegenerative pathway in human diseases and found that the protein levels of Arf1, NeuN and myelination-related proteins were significantly reduced, while the protein levels of IBA1, GFAP, NLRP3, IL-1β, IL-18, activated caspase-1 (p20) and C3 were significantly increased, in brain and spinal cord tissues of ALS and MS patients compared to control persons, suggesting that the Arf1-reduction-induced neuroinflammation pathway might be responsible for some NDs in humans. In this study, we further examined the expression of IFN-γ and IBA1 in postmortem tissues from control persons and patients with ALS and MS and found that both markers in brains from MS and ALS patients were dramatically increased compared to those from control persons (Fig. S12A–C). The synapses were also lost in the brain stem of ALS and MS patients compared to control persons (Fig. S12D and E). These findings demonstrate that the neurodegenerative pathway associated with Arf1 downregulation exists in at least two major NDs, suggesting that it may help to drive neurodegeneration.

## DISCUSSION

Recent studies have revealed that NDs are caused by interactions of multiple cells—including neurons, microglia, astrocytes, oligodendrocytes and immune cells [1–3,29,34]. However, the mechanisms that link these interactions are still unclear. We found that the ablation of Arf1 in neurons caused an accumulation of lipid droplets and peroxided lipids, which then moved to microglia to activate the NLRP3 inflammasome, which released IL-1β; IL-1β together with elevated chemokines recruited and activated γδ T cells in meninges; the activated γδ T cells then secreted IFN-γ that entered into parenchyma to activate the DAM–A1 astrocyte pathway, which released C3 that destroyed neurons via the astrocyte C3-neuronal C3aR1 pathway. Together, our results reveal a neuron-immune circuit of multiple cells that drives neurodegeneration (Fig. S12F).

In the Arf1-ablated mice, there are two types of microglia. The first type of microglia functions upstream of IFN-γ and receives the peroxided lipids from the Arf1-ablated neurons to activate the NLRP3 inflammasome, which expresses IL-1β; the second type of microglia functions downstream of IFN-γ and displays the DAM signatures.

We also found that IFN-γ deficiency almost completely rescued the neurodegenerative phenotypes of the Arf1-ablated mice. IFN-γ produced by meningeal γδ T cells activated microglia through the IFNR-STAT1-mediated signal transduction pathway to induce expression of TNF and IL-1α, which further activated astrocytes to produce C3. It was previously reported that IFNγ produced by meningeal NK cells drives astrocyte activation [35] and IFN-γ produced by meningeal γδ T cells influences mouse behavior [29]. The IFN-γ^+^ NK cells and IFN-γ^+^ γδ T cells performed these functions without infiltrating the CNS parenchyma. Molecules smaller than 40 kDa (such as IFN-γ) have been found in the subarachnoid cerebrospinal fluid (CSF) and may enter CNS parenchyma to influence behavior [29], pain [35], astrocyte-associated anti-inflammation [6], and activation of the reactive microglia-astrocyte pathway, depending on their receptor expression on neurons, astrocytes and microglia, and other specific factors.

Neurotoxic A1 astrocytes are heterogeneous populations and are activated by multiple factors and promote neuronal death through releasing various neurotoxic materials. The neurotoxic materials can be long-chain saturated lipids (LCSLs) [36], protein [37], NO [38], excessive inorganic polyphosphate [39] or complement C3 [40]. In our system, in the neuronal Arf1-ablated mouse brain, IL-1α, IL-1β, TNF and IFN-γ (but not C1q) were upregulated (Fig. S4A–E). We found that IFN-γ selectively activated microglia (Fig. S3A–E). Therefore, we activated astrocytes with IL-1β and TNF and found that the astrocyte C3-neuron C3aR1 pathway, rather than the LCSLs, promoted neuron death.

## METHODS

Detailed methods and materials are provided in the supplementary data of the online version of this paper.

## Supplementary Material

nwad222_Supplemental_File

## Data Availability

Data will be made available on request.
