# Supplementary material for: A neuron-immune circuit regulates neurodegeneration in the hindbrain and spinal cord of Arf1-ablated mice
Source: Natl Sci Rev. 2023 Aug 18;10(12):nwad222. doi: 10.1093/nsr/nwad222 (PMC10794899; doi:10.1093/nsr/nwad222)
Supplement: nwad222_Supplemental_File [file nwad222_supplemental_file.docx]

**Supplementary Material for**

**A Neuron-Immune Circuit Regulates Neurodegeneration in the Hindbrain and Spinal cord of ARF1-Ablated Mice**

**Guohao Wang^2,3^, Shuhan Jin^1,3^, Jiaqi Liu^1^, Xu Li^1^, Peng Dai^1^, Yuetong Wang^1,^ *, and Steven X. Hou^1,2,^ ***

1 Department of Cell and Developmental Biology at School of Life Sciences, State Key Laboratory of Genetic Engineering, Institute of Metabolism and Integrative Biology, Human Phenome Institute, Department of Liver Surgery and Transplantation of Liver Cancer Institute at Zhongshan Hospital, Fudan University, Shanghai 200438, China

2 The Basic Research Laboratory, Center for Cancer Research, National Cancer Institute at Frederick, National Institutes of Health, Frederick, MD 21702, USA

3 These authors made equal contribution

*Correspondence: [ytwang@fudan.edu.cn](mailto:ytwang@fudan.edu.cn) (Y.T.W); stevenhou@fudan.edu.cn (S.X.H.)

**Materials and Methods**

**KEY RESOURCES TABLE**

| **REAGENTS or RESOURA1:C97** | **SOURCE** | **IDENTIFIER** |
| --- | --- | --- |
| **Primary antibodies** |  |  |
| PSD95 | Millipore | MAB1596 |
| Synaptophysin | Synaptic Systems | 101004 |
| IFN-γ | R&D Systems | MAB485-100 |
| IBA1 | Thermo Fisher | PA5-27436 |
| IBA1 | NOVUS Biologicals | NB100-1028 |
| GFAP | Millipore | MAB360 |
| C3 | ALZFORUM | 55444 |
| C3 | Beyotime | AF6357 |
| IgG | Bio X Cell | BE0090 |
| TCRgd | Bio X Cell | BE0070 |
| VLA-4 | Bio X Cell | BE0071 |
| NeuN | Millipore | MAB377 |
| CNPase | Abcam | ab6319 |
| CNPase | Proteintech | Ag4227 |
| Mpz | NOVUS Biological | NB100-1607 |
| Mog | Millipore | MAB5680 |
| Mog | Proteintech | 12690-1-AP |
| Mbp | Millipore | MAB386 |
| Mbp | Proteintech | 10458-1-AP |
| ARF1 | Thermo Fisher | PA1-127 |
| GAPDH | Thermo Fisher | MA5-15738 |
| CD68 | Bio-Rad | MCA1957GA |
| ASC | AdipoGen | AG-25B-0006-C100 |
| STAT1 | Cell Signaling Technology | 9172S |
| STAT1 | Beyotime | AF0288 |
| pSTAT1 | Cell Signaling Technology | 8826S |
| pSTAT1 | Beyotime | AF2212 |
| anti-mouse IL-1β | R&D Systems | AF-401-NA |
| anti-human IL-1β | Novus Biologicals | NB600-633 |
| anti-human IFNγ | R&D Systems | MAB285-100 |
| **Flow cytometry antibodies** |  |  |
| CD16/32 | BioLegend | 101320 |
| CD45-APC/Cy7 | BioLegend | 103116 |
| CD3-Pacific Blue | BioLegend | 100214 |
| CD8a-AF647 | BioLegend | 100724 |
| CD4-PerCP/Cy5.5 | BioLegend | 116012 |
| TCRb-FITC | BioLegend | 109206 |
| TCRgd-PE | BioLegend | 118107 |
| NK1.1-BV650 | BioLegend | 108735 |
| CD25-BV785 | BioLegend | 102051 |
| CD127-PE/Cy7 | BioLegend | 135013 |
| CD11b-BV421 | BioLegend | 101251 |
| Ly-6G/C-FITC | BioLegend | 108405 |
| F4/80-BV650 | BioLegend | 123149 |
| CD11c-BV785 | BioLegend | 117336 |
| B220-PE | BioLegend | 103208 |
| MHC-II-PerCP/Cy5.5 | BioLegend | 107625 |
| CD1d-BV711 | BD Biosciences | 740711 |
| Foxp3-BV421 | BD Biosciences | 562996 |
| **Chemicals peptides, and recombinant proteins** |  |  |
| BODIPY 493/503 | Thermo Fisher | D3922 |
| BODIPY C11 | Thermo Fisher | D3861 |
| Hoechst | Thermo Fisher | H1399 |
| MCC950 | InvivoGen | inh-mcc |
| α-Bungarotoxin | Thermo Fisher | B35451 |
| Tamoxifen | Sigma-Aldrich | T5648 |
| Adenosine 5'-Triphosphate (ATP) | New England Biolabs | P0756S |
| minocycline | Sigma-Aldrich | PHR1801 |
| Oil Red O | Sigma-Aldrich | O0625 |
| N-acetylcysteine amide (AD4) | R&D Systems | 5619/50 |
| oxATP | Sigma-Aldrich | A6779 |
| GCA | Cayman Chemical | 18430 |
| BFA | Thermo Fisher | B7450 |
| Cleanert LipoNo | Agela Technologies | MS-LN0415 |
| Mouse TNF(aa 80-235) | R&D Systems | 410-MT-050/CF |
| Mouse IL-1β/IL-1F2 | R&D Systems | 401-ML-025/CF |
| Mouse C3 | CompTech | M113 |
| C3 Fusion Protein | proteintech | Ag15955 |
| **Experimental models: Cell lines** |  |  |
| N2a | ATCC | CCL-131^™^ |
| EOC20 | ATCC | CRL-2469R^™^ |
| C8D1A | ATCC | CRL-2541^™^ |
| LADMAC | ATCC | CRL-2420 |
| HEK293 | ATCC | CRL-1573 |
| **Critical commercial assays** |  |  |
| RBC Lysis Buffer | Beyotime | Cat# C3702 |
| RIPA Lysis Buffer | Beyotime | Cat# P0013B |
| CCK-8 Cell Proliferation and Cytotoxicity Assay Kit | Solarbio | Cat# CA1210 |
| Pierce™ BCA Protein Assay Kit | Thermo Fisher Scientific | Cat# 23227 |
| ATP Assay kit | Beyotime | Cat# S0026 |
| ENLITEN® ATP Assay System | Promega | FF2000 |
| ELISA MAX™ Deluxe Set mouse IL-1α | BioLegend | 433404 |
| Mouse IL-1β ELISA Kit | BioLegend | 432601 |
| Mouse IFNγ ELISA Kit | BioLegend | 430801 |
| TNF alpha Mouse ELISA Kit | Invitrogen | BMS607-3 |
| Mouse Complement C3 ELISA Kit | MyBioSource | MBS763294 |
| Lipid Peroxidation (MDA) Assay Kit (Colorimetric/Fluorometric) | Abcam | ab118970 |
| RNeasy Micro Kit | Qiagen | 74104 |
| High-Capacity cDNA Reverse Transcription Kit | Applied Biosystems | 4368814 |
| SYBR™ Select Master Mix | Applied Biosystems | 4472903 |
| **Plasmids** |  |  |
| sh-Scram | Addgene | 1864 |
| sh-Arf1 | Sigma-Aldrich | TRCN0000100371  TRCN0000100373 |
| sh-GFP | Santa Cruz Biotechnology | sc-45924-V |
| **Experimental models: Organisms/strains** |  |  |
| Mouse: C57BL/6J | GemPharmatech | Strain #: N00013 |
| Mouse: Rag1^-/-^ | The Jackson Laboratory | Strain #: 002216 |
| Mouse: IFN-γ^-/-^ | The Jackson Laboratory | Strain #: 002287 |
| Mouse: NLRP3^-/-^ | The Jackson Laboratory | Strain #: 021302 |
| Mouse: TLR4^-/-^ | The Jackson Laboratory | Strain #: 029015 |
| Mouse: C3^-/-^ | The Jackson Laboratory | Strain #: 003641 |
| Mouse: Thy1-CreER (SLICK-H) | The Jackson Laboratory | Strain #: 012708 |

**EXPERIMENTAL MODEL AND SUBJECT DETAILS**

**Mice**

All mice were bred in the animal facility at the National Cancer Institute (NCI) at Frederick under specific pathogen-free conditions in a temperature-controlled environment with a 12-hour day/night light cycle. All breeding and maintenance were handled in accordance with the guidelines of the Animal Care and Use Committee of NCI, National Institutes of Health (NIH). The *Arf1*-floxed mice were generated in the animal core facility of the Mouse Cancer Genetics Program at NCI, as described previously (Wang et al., 2020). Rag1^-/-^ (002216), IFN-γ^-/-^ (002287), NLRP3^-/-^ (021302), TLR4^-/-^ (029015), C3^-/-^ (003641), Thy1-CreER (SLICK-H) (012708) mice were obtained from The Jackson Laboratory. To generate *Arf1*-knockout mice in neurons, we crossed homozygous floxed Arf1 mice with Thy1-CreER transgenic mice that express tamoxifen-inducible Cre in neurons. Double-knockout of *Arf1* with *IFN-γ*, *Rag1*, *NLRP3*, or *TLR4* was generated by crossing *Thy-1-CreER/Arf1^f/f^* mice with IFN-γ^-/-^, Rag1^-/-^, NLRP3^-/-^, C3^-/-^ or TLR4^-/-^ mice for three generations to get the homozygous mice. Primers were used for genotyping mice as provided by The Jackson Laboratory website. Arf1 genotyping primers were used as follows: Arf1-gP5, 5’-GGTTTTAAGAGGCCCTGTGTC-3’; Arf1-gP3’, 5’-TCGGGAGCTGGCACTAAAAA-3’; and Arf1-gR, 5’- TGCACACACCAAGTACAAGC-3’.

**Injecting chemicals and proteins into mouse**

Tamoxifen (T5648) was dissolved in corn oil (Sigma-Aldrich, C8267) and intraperitoneally (i.p.) injected into mice at 0.01 mg/g of body weight for five consecutive days. For treatment of mice with minocycline, MCC950, AD4, and oxATP, minocycline was dissolved in saline and injected i.p. at 50 mg/kg each day for two consecutive weeks; the MCC950 was dissolved in phosphate-buffered saline (PBS), and the mice were injected i.p. with MCC950 (10 mg/kg) or PBS once every two days for two consecutive weeks; AD4 and oxATP were dissolved in PBS, and AD4 (100 mg/kg) and oxATP (15 mg/kg) were administered to each mouse by i.p. injection at 24-hour intervals for two consecutive weeks, beginning 24 hours after mice were challenged with tamoxifen five times.

To administe TNF (50 ng), IL-1β (100 ng), and/or C3 (1 μg) into the spinal cord of adult mice weighing 22.0-26.0 g per mouse, the mice were initially anesthetized with 3% isoflurane. Once the mice were fully asleep, the proteins dissolved in 100 mL of saline were injected between the L5-L6 spinal cord level using a 30G needle. After the protein administration, the mice were kept in their home cages, mice were euthanized after 1 week for subsequent biochemistry and immunohistochemistry analysis.

**Mouse behavior test**

Mouse body weight was measured daily by using a balance after tamoxifen injection. Fine motor coordination and balance were assayed by balance beam test. Mice were trained for two days to walk the entire length of a 6-mm- or 12-mm-wide × 80-cm-long wooden beam suspended 50 cm above the floor. The time for a mouse to cross the beam was recorded. In each test, the mice were placed onto one end of the beam and ran down the entire beam into a dark box. Each genotype used five mice, and each mouse was tested three times. The results were the average of 15 trials.

Neurological score was observed daily with disease development after tamoxifen injection. Throughout disease progression, mice were scored to indicate neurological damage, as follows: 0 – no clinical signs; 1 – limp tail or hindlimb weakness; 2 – limp tail and hindlimb weakness; 3 – loss of coordinated movements; 4 – hindlimb paralysis; 5 – hindlimb and forelimb paralysis; 6 – moribund.

**Single cell RNA-seq**

Microglia cells were sequenced by four 10x Genomics Chromium Single Cell platform. The barcoded libraries were generated following the manufacturer’s specifications, and 3’ mRNA libraries were made and sequenced on one NextSeq run and one NovaSeq SP run. All samples have sequencing yields of more than 315 million reads per sample. The sequencing was set up as a 28 cycles + 55 cycles non-symmetric run on the NextSeq and as a 28 cycles + 75 cycles non-symmetric run on the NovaSeq. Demultiplexing was done, allowing one mismatch in the barcodes. Over 97.2% of bases in the barcode regions have Q30 or above, at least 95.0% of bases in the RNA read have Q30 or above, and 95.6% or more of bases in the sample index have Q30 or above. More than 97.2% of bases in the unique molecular identifier (UMI) have Q30 or above.

The analysis was performed by the Center for Cancer Research Collaborative Bioinformatics Resource at NCI using the default parameters and aligned to the GRCm38 (mm10) mouse reference genome. The number of captured cells ranges from 27,930–30,710, and mean reads per cell ranged from 10,611–13,205. Cells with an extremely low number of UMI counts were filtered out. Median genes found per cell ranged from 1,111–1,187, and the total number of genes detected ranged from 23,003–23,633. Morethan 50% cells were recognized as microglia. Uniform Manifold Approximation and Projection (UMAP) was generated to create a map of merged cells from four samples of mouse brain. Tabula Muris was used to annotate cell identities. Microglia were extracted and retransformed for all subsequent analysis. Microglia were separated into 12 clusters. Model-based analysis of single cell transcriptomics (MAST) was used to compute differentially expressed genes for each cluster compared to the rest of the clusters. Genes with an adjusted p value of 0.05 or below and an absolute log2 fold change of 0.5 or above were annotated as significant. Chi-squared test was used test for any disproportional distribution of cell numbers in each cluster. Signed value was calculated for each gene as Signed.value = sign (logFC) * log10 (P-value) * abs (log FC). Genes that fell on the top right quadrant indicate concordant up-regulation with previously identified disease state genes. Genes that fell on the bottom left quadrant indicate concordant down-regulation with previously identified disease state genes. DEGs from both Arf1-WT contrast and Cluster5-ClusterAll contrast showed a high number of genes that showed similar signed value signatures as those disease state genes. We computed Cluster5-cluster1 differentially expressed genes and performed the over- representation analysis using the Gene Ontology – Biological Process gene set.

**Electron microscopy**

For electron microscopy, adult mice were euthanized with CO_2_ for 30 minutes, and the brain and spinal cord were isolated using surgical scissors and tweezers. The brain and spinal cord were perfused with electron microscopy buffer containing 4% paraformaldehyde (PFA), 0.2% glutaraldehyde, and 0.1 M sodium cacodylate for 48 hours. Fixed tissues were sent to the NCI Electron Microscopy Laboratory at the Advanced Technology Research Facility in Frederick, Maryland, for sectioning and imaging.

**Western blot**

Mouse brains, spinal cords tissues, or cells were homogenized in RIPA lysis buffer (0.1% SDS, 150 mM NaCl, 0.5% sodium deoxycholate, 1 mM EDTA, 1 mM EGTA, 1.0% Triton X-100, and 50 mM Tris pH 8.0) with 1 × phosphatase inhibitor cocktail (Sigma-Aldrich) and 1 × protease inhibitor cocktail (Promega) 30 times and lysed on ice for 30 minutes, sonicated at 20 W for 30 seconds, and then centrifuged at 12,000 × *g* at 4 °C for 2 minutes to delete pellets. The saved supernatants were used for gel analysis. The protein concentration was determined by a Bio-Rad protein assay kit (Bio-Rad). A total of 300 µL of protein (2.5 µg/µL) was added with 1 × SDS loading buffer and boiled by a 100 °C heater for 15 minutes. Equal volumes of protein samples were loaded onto 4–20% Mini-PROTEAN® TGX™ precast protein gels (Bio-Rad) and run by a 120 V PowerPac™ Basic power supply (Bio-Rad) to electrophorese for 65 minutes. The gel was transferred on nitrocellulose membrane (GE Healthcare). After transfer, the membrane was blocked with 5.0% blotting-grade blocker (Bio-Rad) for 1.5 hours, and then incubated with primary antibodies at 4 °C with shaking overnight. The next day, the membrane was washed three times with 1 × PBST for 5 minutes each and incubated with the secondary HRP antibodies (1:2000) in 5% milk for 2 hours. The membrane was then developed by Pierce™ ECL Western Blotting Substrate (Thermo Fisher, 32106), applied to HyBlot CL® autoradiography film (Thomas Scientific, 3022), and developed with an S&W Imaging system (Quantum Medical Enterprises LNC.).

**RNA isolation and qRT-PCR**

Total RNA of mouse brains or spinal cords was extracted with the RNeasy Micro Kit (Qiagen, 74104), and cDNA was synthesized by the High-Capacity cDNA Reverse Transcription Kit (Applied Biosystems, 4368814). RNA concentration was determined by a NanoDrop (DS-11 spectrophotometer from DeNovix, Inc.), and an equal amount of RNA was used for cDNA synthesis. The cDNA amplification was performed with a SYBR™ Select Master Mix (Applied Biosystems, 4472903) using a CFX96 Touch™ Real-Time PCR Detection System (Bio-Rad). The *Gapdh* gene was used as an internal control. The results are shown as 2^−ΔCT^. Each sample underwent three independent experiments. The pan-astrocyte, A1 astrocyte, and A2 astrocyte primer sequences were used as described previously (Yun et al., 2018). The primers used for C3aR1 QPCR are: C3aR1-F: TCGATGCTGACACCAATTCAA, C3aR1-R: TCCCAATAGACAAGTGAGACCAA.

**Cell culture**

Mice Neuro-2a and LADMAC cells were purchased from American Type Culture Collection (ATCC) and cultured in Eagle’s minimum essential medium supplemented with 10% fetal bovine serum (FBS), 100 U/mL penicillin, and 100 µg/mL streptomycin. HEK 293 cells were cultured in Dulbecco’s modified Eagle’s medium (DMEM) supplemented with 100 µg/mL streptomycin, 100 U/mL penicillin, and 10% FBS. EOC 20 mouse microglia cells were purchased from ATCC and cultured in DMEM containing 4 mM L-glutamine adjusted to contain 1.5 g/L sodium bicarbonate and 4.5 g/L glucose, with 10% FBS and 20% LADMAC conditioned media (produced from the LADMAC cell line). C8D1A mouse astrocyte cells were purchased from ATCC and cultured in DMEM supplemented with 100 µg/mL streptomycin, 100 U/mL penicillin, and 10% FBS.

**shRNA knockdown**

For knockdown C3aR1, we used lenti-virus infection system. The shRNA sequence are listed below. shC3aR1-F-1::CCGGGCAGCCAGATTGGCTCTTATTCTCGAGAATAAGAGCCAATCTGGCTGCTTTTTG; shC3aR1-R-1:AATTCAAAAAGCAGCCAGATTGGCTCTTATTCTCGAGAATAAGAGCCAATCTGGCTGC; shC3aR1-F-2:CCGGCCTGTATACATCGAAGGATAACTCGAGTTATCCTTCGATGTATACAGGTTTTTG; shC3aR1-R-2:AATTCAAAAAGCCTTGTGTCATGGCTTCAATCTCGAGATTGAAGCCATGACACAAGGC. HEK293T cells were seeded in 6-well plates in D10 media. The following day, 3 μL TransIT-LT1 transfection reagent (Mirus Bio) wasmixed with 15 μL Opti-MEM (Thermo) and incubated for 5 min at room temperature (RT). A mixture of 500 ng expression plasmid, 500 ng packaging plasmid psPAX2 (Addgene #12260), and 250 ng envelope plasmid pMD2.G (Addgene #12259) was prepared to a ﬁnal volume of 37.5 μL in Opti-MEM. Transfection reagent mix was combined with plasmid mix, incubated for 30 min at RT, and then added dropwise to HEK293T cells. After 24 h, cell media was replaced with fresh medium. Cell media was collected and replaced after 48 h and 72 h and ﬁltered through a 0.45 μm syringe ﬁlter (ThermoFisher). Then the N2A cells were infected with collected medium.

**Culture of primary mouse neurons and astrocytes**

Primary culture of mouse hindbrain neurons was performed as described previously [34]. Pregnant female mice with E17.5–18.5 embryos were anesthetized and embryos were isolated from the uterus. The hindbrain was dissected, placed in ice-cold HBSS and digested with papain solution at 37 °C for 30 min. Brain lysates were centrifuged for 5 min at 800 r.p.m. at room temperature. The cell pellet was resuspended in Hank’s solution containing DNase I and then dissociated into single cells by gentle pipetting up and down using a 1 mL pipette. After that, cells were transferred into Hank’s solution containing 10 mg/mL trypsin inhibitor (Sigma, T9253) and 10 mg/mL BSA (Sigma, A9647). Cells were centrifuged at 800 r.p.m. for 10 min and resuspended in neurobasal medium (2% fetal bovine serum (Gibco, 10437028), 2% B27 supplements (Thermo Fisher, 17504044)) and 2 mM L-glutamine (Thermo Fisher, 25030149). Neurons at a density of 0.8–0.9 × 105 cells per well were plated on 12-mm glass coverslips residing in 24-well plates that were coated with poly-d-lysine (Sigma, P6407). The neuron medium was changed by a half volume once a week, and included 2% B27 neurobasal maintenance medium (GIBCO, 17504044) supplements and 2 mM L-glutamine.

The details of primary culture of mouse brain astrocytes cells were described previously^35,36^. Cortical astrocytes were prepared from newborn mice. Cortices were cut into small cubes (<1 mm^3^) and digested with 0.25% trypsin for 30 min at 37℃. Trypsinization was terminated by the addition of Dulbecco’s modified Eagle’s medium (Gibco) containing 10% fetal bovine serum, followed by mechanical trituration with a flame- polished Pasteur glass pipette. Cell suspensions were sieved through a 40 μm cell strainer. The filtrate was allowed pre-adherence for 30 min to remove any contamination from fibroblasts before being seeded at a density of 1 × 10^6^ cells/cm^2^ in 50 cm^2^ flasks (Corning) or in six-well plates. The plated cells were cultured in a 5% CO_2_ incubator at 37℃ for 7 days and the culture medium was changed at 3-day intervals. When cells grew to confluence, flasks were shaken on a rotary shaker at 260 rpm for 18–20 h at 37 °C to remove the loosely attached contaminated microglia and OPCs. The attached enriched astrocytes were subsequently detached using trypsin-EDTA and then subjected to next experiments.

**Immunohistochemistry and Immunofluorescence**

Mice were anesthetized by Isothesia isoflurane (Henry Schein, NDC11695-6776-2) perfused with 20 mL saline and further perfused with 4% PFA in 0.01 M PB. Brains and spinal cords were isolated and fixed by 4% PFA overnight and transferred into 30% sucrose at 4 °C to let the tissues completely sink to the bottom of the tube. The tissues were sectioned with paraffin embedding at 10 μm or frozen sectioning at 20 μm. The sections were deparaffinized with xylene and alcohol, and antigen retrieval was performed by citrate buffer (Abcam, ab93678) or Tris-EDTA buffer (Abcam, ab93684) in a 100 °C cooker for 30 minutes. Mouse brain and spinal cord slides or cultured cells were fixed with 4% PFA at room temperature for 15 minutes, washed three times with 1 × PBST, blocked with 10% goat serum for 1 hour, and incubated overnight with primary antibodies in 3% bovine serum albumin at 4 °C. The next day, the slices were washed three times with 1 × PBS and incubated with Hoechst and secondary antibodies (AF-488, 594, or 647; Thermo Fisher) for 2 hours at room temperature. The cells or sections were examined by using a Zeiss (LSM780) confocal microscope system and analyzed with Zeiss black software.

**Flow cytometry analysis**

Mice meninges and brain were isolated as described previously[41]. Cell surface markers were stained with relative antibodies (purchased from BioLegend and BD Biosciences Ltd.) for 30 minutes at 4 °C in the dark. (The details of the methods performed are described on the BioLegend website.) For staining of the intranuclear transcription factor Foxp3, cells were stained with surface marker and fixed with 0.5 mL/tube Fixation Buffer (BioLegend, 422101) in the dark for 20 minutes at room temperature according to the manufacturer’s instructions. Then, cells were washed twice with 1 mL of Intracellular Staining Permeabilization Wash Buffer and centrifuged at 350 x *g* for 5 minutes. The supernatant was discarded, and the cells were stained with intercellular antibody. Stained cells were analyzed with a BD LSRFortessa cell analyzer, and the data were analyzed with FlowJo 10 software.

**Enzyme-linked immunosorbent assays (ELISA)**

The mouse extracellular ATP, IL-1α, IL-1β, IFN-γ, and C3 analyses were performed with ELISA kits purchased from commercial vendors, including the ENLITEN® ATP Assay System (Promega, FF2000), ELISA MAX™ Deluxe Set mouse IL-1α (BioLegend, 433404), Mouse IL-1β ELISA Kit (BioLegend, 432601), Mouse IFN-γ ELISA Kit (BioLegend, 430801), TNF alpha Mouse ELISA Kit (Invitrogen, BMS607-3), Mouse Complement C3 ELISA Kit (MyBioSource, MBS763294), and Lipid Peroxidation (MDA) Assay Kit (Colorimetric/Fluorometric) (Abcam, ab118970). Detailed methods for performing the analysis are described in the manufacturers’ instructions.

**Treatment Mice with Neutralizing Antibody**

Rat IgG1 isotype control (BP0088) and neutralizing antibodies of IFNγ (BP0055), VLA-4 (BE0071), TCRg/d (BE0070), and Armenian hamster IgG (BE0091) were purchased from Bio X Cell Ltd. Each mouse was i.p. injected with neutralizing antibody of IFN-γ (250 μg/mouse), TCRg/d (400 μg/mouse), and VLA-4 (300 μg/mouse) or relative amounts of control antibodies every two days for three weeks. The mice injected with control antibodies and PBS had similar phenotypes.

**Human samples**

Human postmortem brain and spinal cord tissue samples were obtained from the NIH NeuroBiobank, the University of Maryland Brain and Tissue Bank, and the Rocky Mountain Multiple Sclerosis Center Tissue Bank. All experimental procedures followed the NIH NeuroBiobank’s guidelines and restrictions. Age-matched control human tissues were used for comparison with MS and ALS patients’ tissues. Each group used three independent human tissues. Human sample information is described in **Supplemental Table 2**.

**Statistical analysis**

Each group in the mouse behavior study used at least five mice. The number of mice used is shown in the figure legend; the cell experiments were performed with triplicate samples and in two independent experiments. Data are shown as mean ± SEM or mean ± SD. Comparisons of two groups were done by paired two-tailed Student’s t-test. Analysis of more than two groups was performed by one-way or two-way ANOVA (with Bonferroni multiple-comparison post-tests). Statistical analysis was done with GraphPad Prism 9. A P value < 0.05 was considered significant.

**Ethics approval and consent to participate**

Human postmortem brain and spinal cord tissue samples were obtained from the NIH NeuroBiobank, the University of Maryland Brain Tissue Bank, and the Rocky Mountain Multiple Sclerosis Center Tissue Bank. All experimental procedures followed the NIH NeuroBiobank’s guidelines and restrictions. Age-matched control human tissues were used for comparison with MS and ALS patients’ tissues. Each group used three independent human tissues. Human sample information is described in Supplemental Table 2.


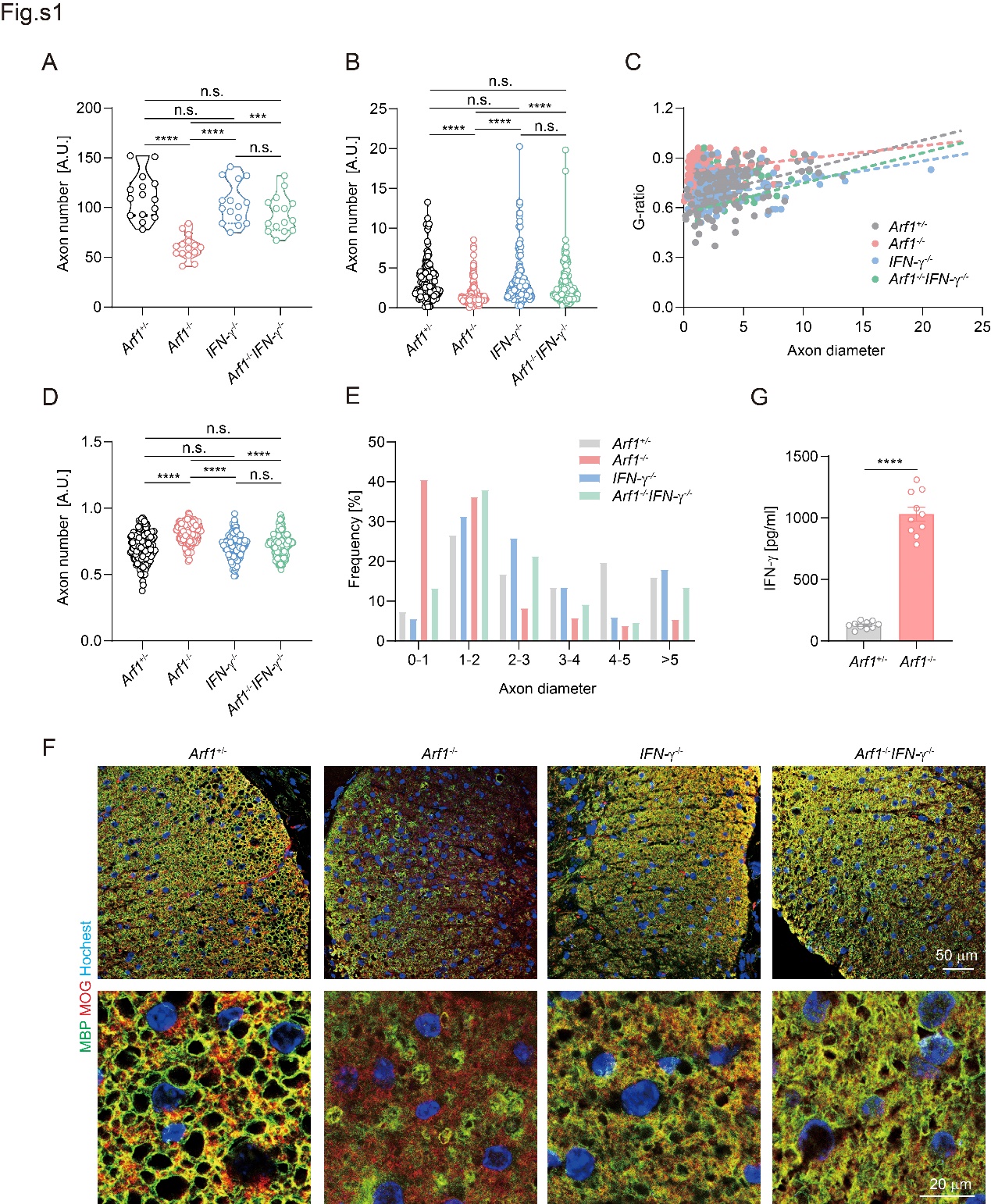


**Supplementary Fig. S1 ARF1 ablation promotes neurodegeneration through IFNγ.**

(A-E) Axon degeneration phenotypes associated with ARF1-ablated mice were suppressed in IFN-γ deficient background. Mean axonal numbers (A), mean axonal diameters (B), individual G-ratio distribution (C), mean G-ratios (D), and distributions of axonal diameters (E) in the ventrolateral lumbar spinal cord white matters of *Arf1^+/–^*, *Arf1^–/–^,* *IFN-γ^-/-^*, and *Arf1^-/-^IFN-γ^-/-^* mice as indicated. Data are shown as Mean ± SEM. n.s. meas no significant difference, **P < 0.01, ***P < 0.001, ****P<0.0001 using one-way (A, B, and D) or two-way ANOVA (C) with Bonferroni multiple comparison test. .

(F) Immunofluorescence staining for Mbp, Mog and Hoechst revealed that IFN-γ deficiency suppressed reduction of myelin proteins in the spinal cord white matters of *Arf1*-ablated mice. Scale bar: 50 μm (top), 20 μm (bottom).

(G) Assay of the IFN-γ level by ELISA in the mice spinal cord lysates after *Arf1* knockout 10 days. n = 10 mice per group, ****P<0.0001 using t-test.


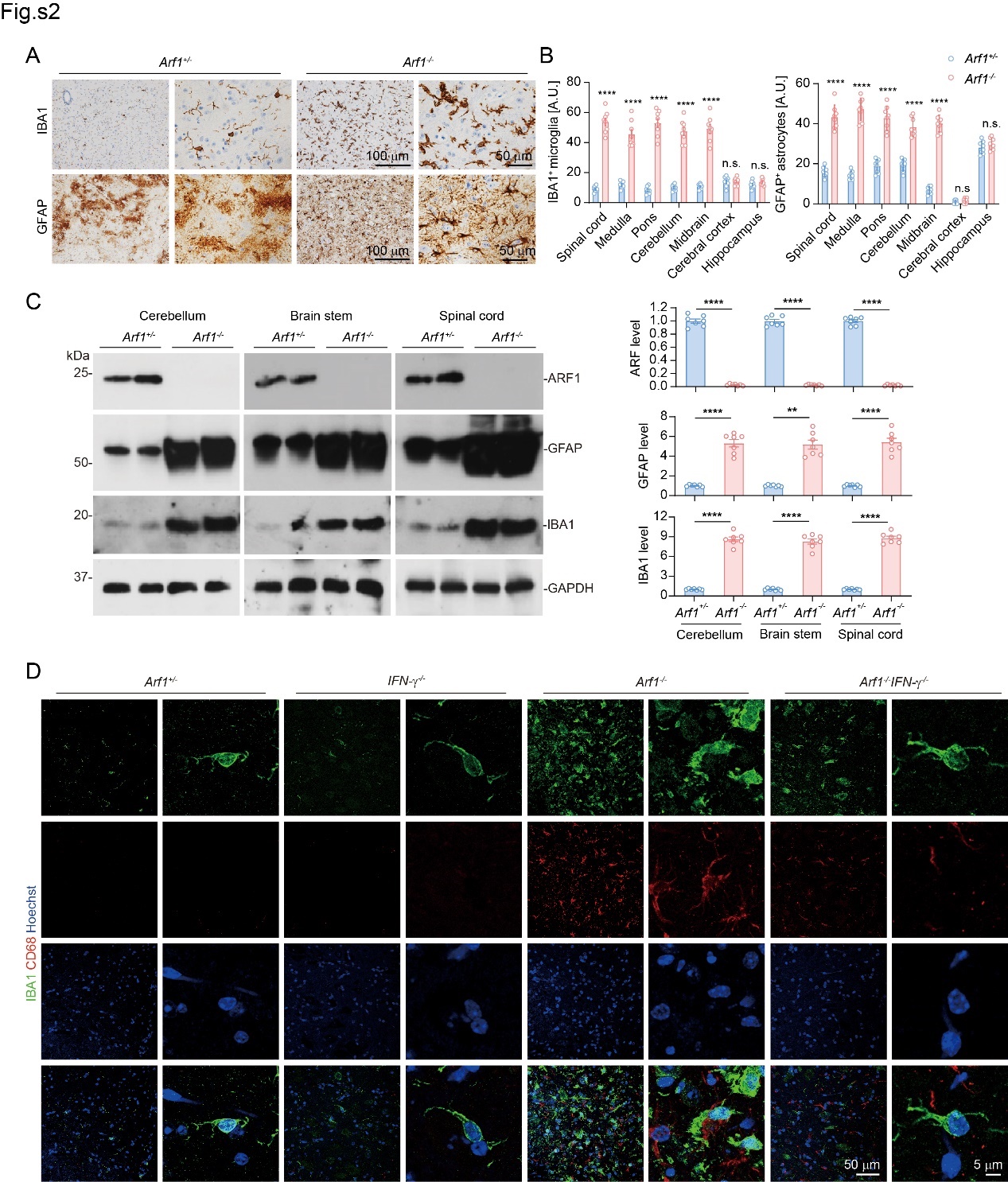


**Supplementary Fig. S2 The reactive microglia-astrocyte pathway is activated in ARF1-ablated mice.**

(A) Representative spinal cord sections from mice with the indicated genotypes were analyzed by immunohistochemistry for IBA1 (microglia) and GFAP (astrocytes).

(B) Quantification of IBA1^+^ microglia and GFAP^+^ astrocytes in different brain areas of mice with the indicated genotypes (n = 8 in each group).

(C) Western blot detection of ARF1, GFAP, IBA1, and GAPDH in lysates of cerebellums, brain stems, and spinal cords from mice with the indicated genotypes (n = 7 per group).

(D) Expression of microglial markers IBA1 and CD68 in the spinal cords of mice with indicated genotypes. Scale bar: 50 μm (left), 5 μm (right).

Data are shown as Mean ± SEM. n.s. meas no significant difference, *P<0.05, **P < 0.01, ***P < 0.001, ****P<0.0001 using two-way ANOVA (B and C) with Bonferroni multiple comparison test.


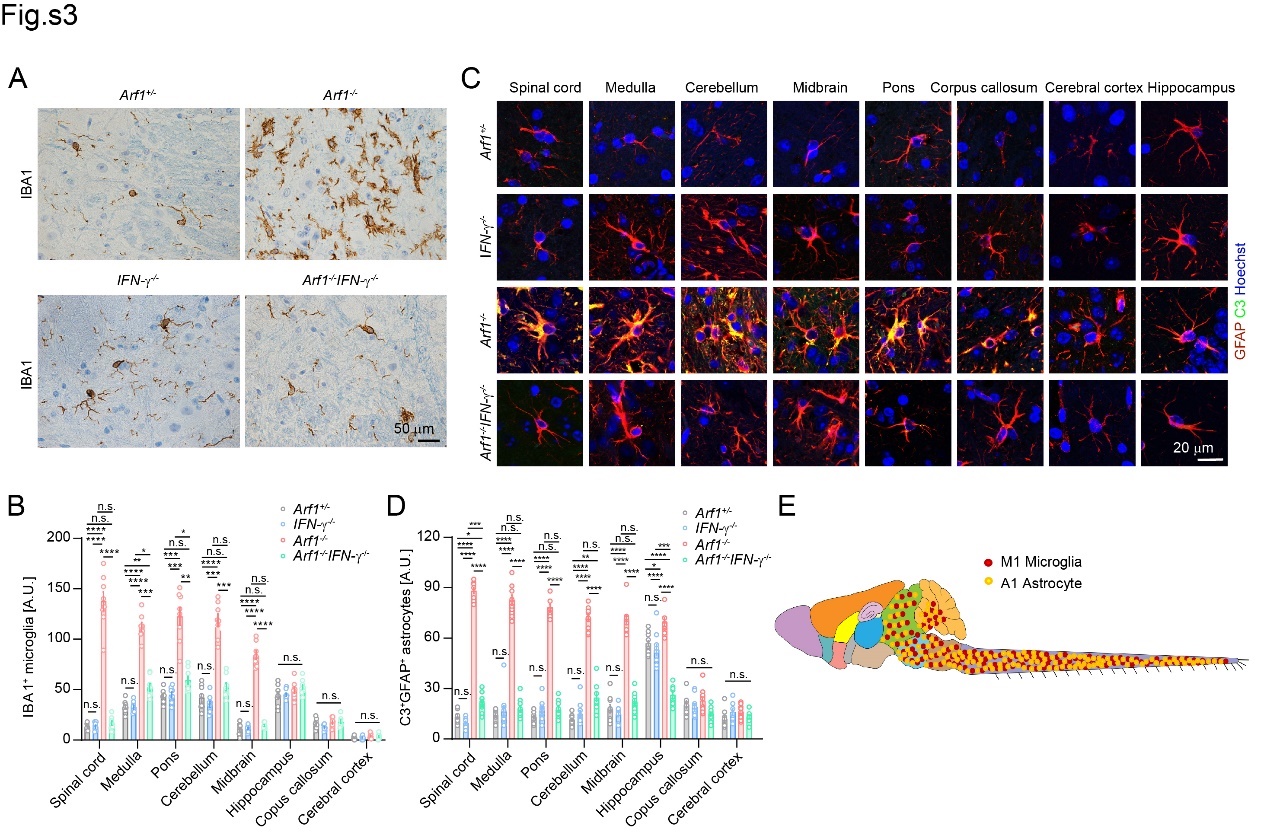


**Supplementary Fig. S3 IFN-γ deficiency almost completely suppressed the reactive microglia and astrocyte phenotypes of ARF1-ablated mice.**

(A) Representative spinal cord sections from mice with the indicated genotypes were analyzed by immunohistochemistry for IBA1 (microglia). Scale bar: 50 μm.

(B) Quantification of IBA1^+^ microglia in different brain areas of mice with the indicated genotypes (n = 8 per group).

(C) Immunofluorescence staining for GFAP and C3 in spinal cords from mice with the indicated genotypes. Scale bar: 10 μm.

(D) Quantification of GFAP^+^ C3^+^ astrocytes in different brain areas of mice with the indicated genotypes (n = 8 per group).

(E) Schematic map of brain domains with activated M1 microglia and A1 astrocytes in ARF1-ablated mice.

Data are shown as Mean ± SEM. n.s. meas no significant difference, *P<0.05, **P < 0.01, ***P < 0.001, ****P<0.0001 using two-way ANOVA (B and D) with Bonferroni multiple comparison test.


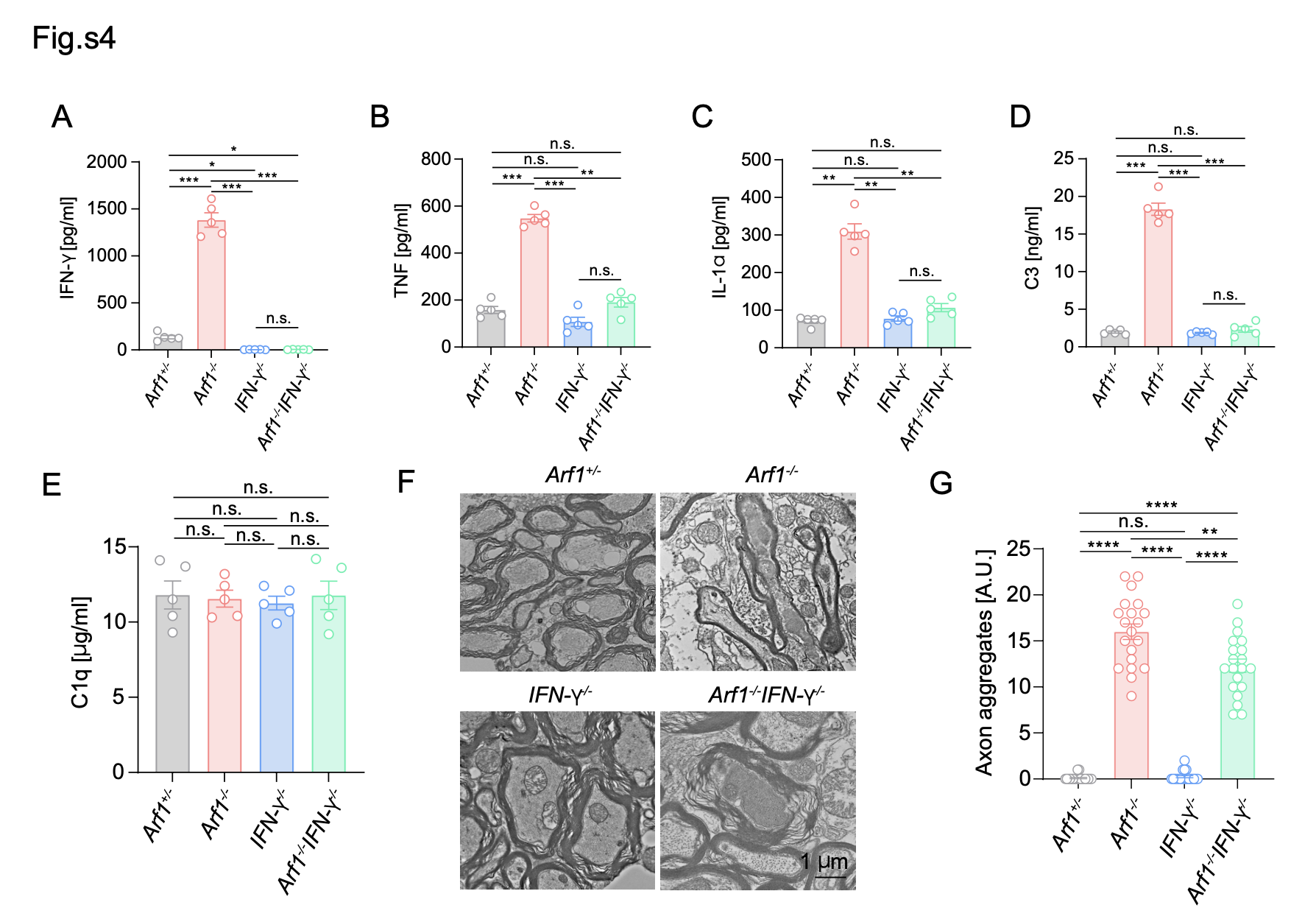


**Supplementary Fig. S4 IFN-γ deficiency almost completely suppressed the major neurodegenerative phenotypes of ARF1-ablated mice but did not affect ARF1-ablation-induced lipid peroxidation.**

(A–E) IFN-γ deficiency almost completely suppressed the neurodegenerative phenotypes of Arf1-ablated mice, including induction of IFN-γ (A), TNF (B), IL-1α (C), and C3 (D). C1q expression is not affected by ARF1 and IFN-γ deficiencies (E). n = 5 per genotype, representing one of three independent experiments.

(F) Electron microscopy sections of mouse spinal cords with the indicated genotypes.

(G) Quantification of axon aggregates in the spinal cords of mice with the indicated genotypes (n = 20 images per genotype from 5 mice).

n.s. meas no significant difference, *P<0.05, **P < 0.01, ***P < 0.001, ****P<0.0001 using one-way ANOVA with Bonferroni multiple comparison test.


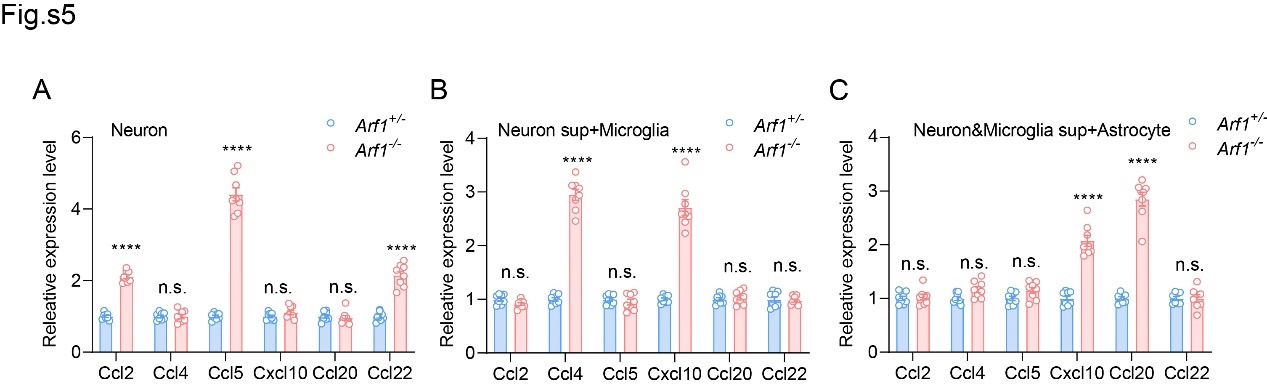


**Supplementary Fig. S5 Chemokines secreted by neuron and neuronal-activated microglia.**

(A-C) Expression of chemokines in cultured neuron (A) with ARF1-ablated (*Thy-1-CreER/Arf1^f/f^*, Arf1^-/-^) or without control (*Thy-1-CreER/Arf1^f/+^*, *Arf1^+/-^*) knockdown, in microglia (B) cultured with supernatants from neuron with (*Arf1*^-/-^) or without (*Arf1*^+/-^) knockdown, in astrocyte (C) cultured with supernatants from neuron + microglia co-culture with (*Arf1*^-/-^) or without (*Arf1*^+/-^) neuronal ARF1 knockdown (n = 8 per group).

n.s. meas no significant difference, *P<0.05, **P < 0.01, ***P < 0.001, ****P<0.0001 using two-way ANOVA with Bonferroni multiple comparison test.


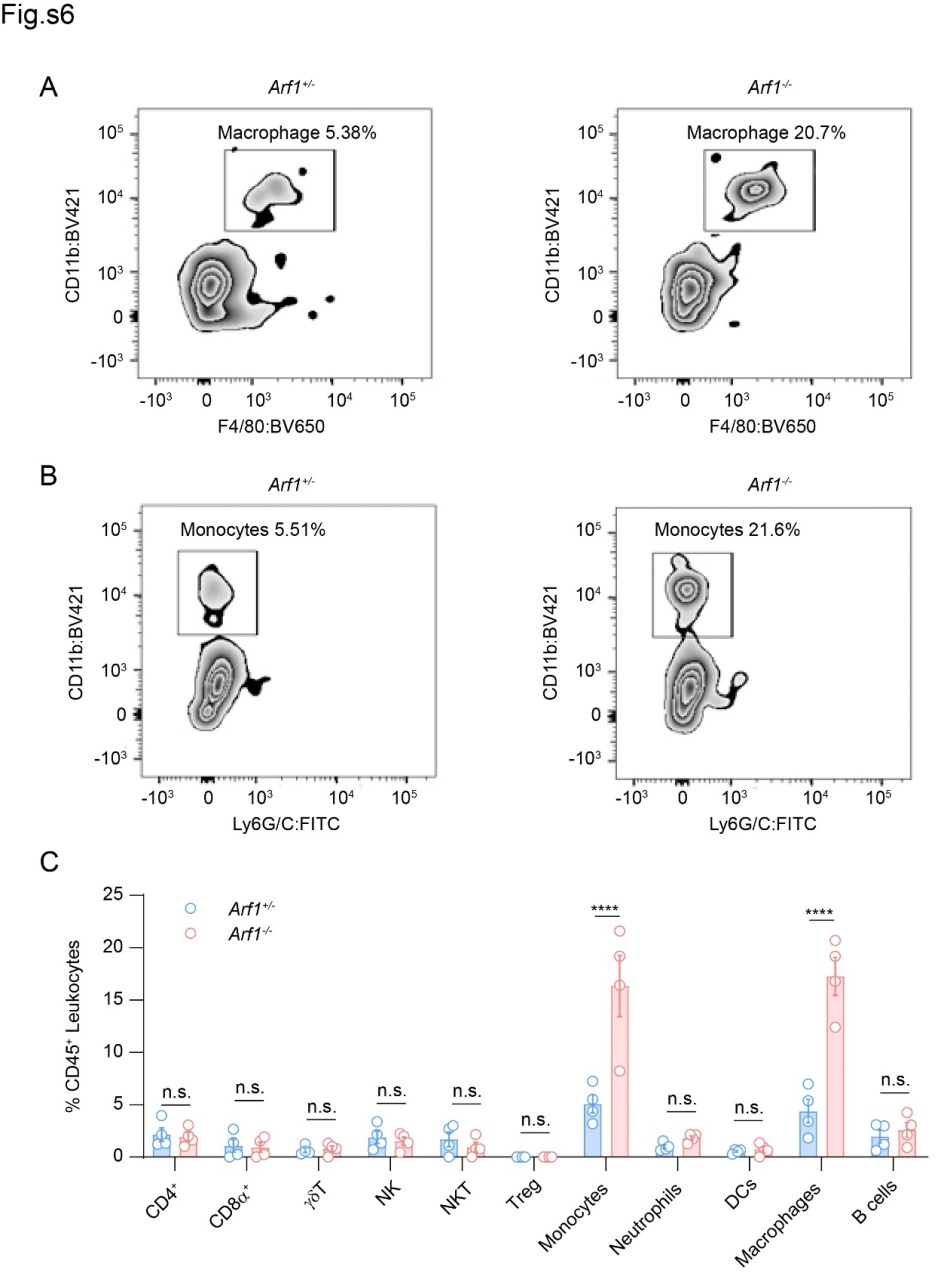


**Supplementary Fig. S6 ARF1 knockout does not significantly affect T and B cells in brain parenchyma.**

(A, B) Representative flow cytometry plots showing frequencies of CD11b^+^F4/80^+^CD45^+^ monocytes and CD11b^+^Ly6G/C^+^CD45^+^ macrophages in cerebellum and spinal cord tissues of control and ARF1-ablated mice.

(C) Bar graph showing frequencies of immune cells in the spinal cord and cerebellum of control and ARF1-ablated mice (n = 4 per genotype).

n.s. meas no significant difference, ****P<0.0001 using two-way ANOVA with Bonferroni multiple comparison test. .


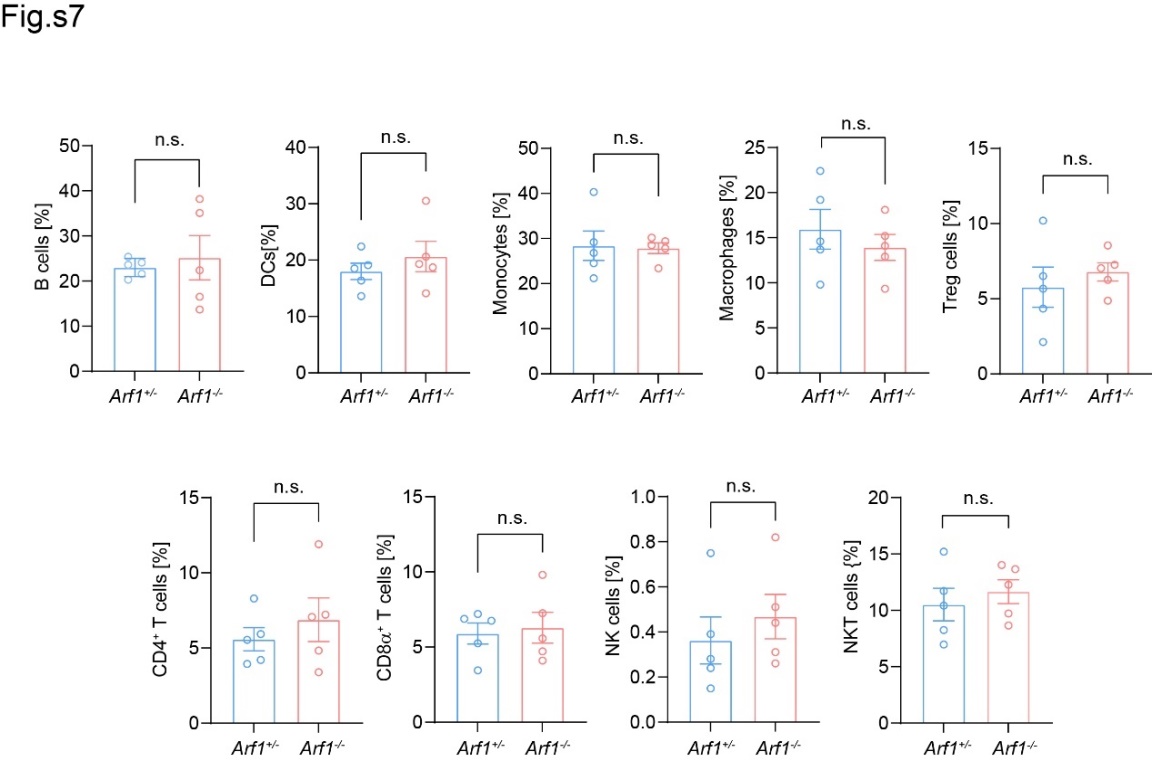


**Supplementary Fig. S7 ARF1 knockout only affects γδ T cells in meninges.**

Meninges were dissected, and single-cell suspensions were immunostained with immune-cell-specific makers for sorting and counting by flow cytometry. No significant changes were found in the indicated cell types between control and ARF1-ablated mice (n = 5 per group). n.s. meas no significant difference, *P<0.05, **P < 0.01, ***P < 0.001, ****P<0.0001 using unpaired t-test.


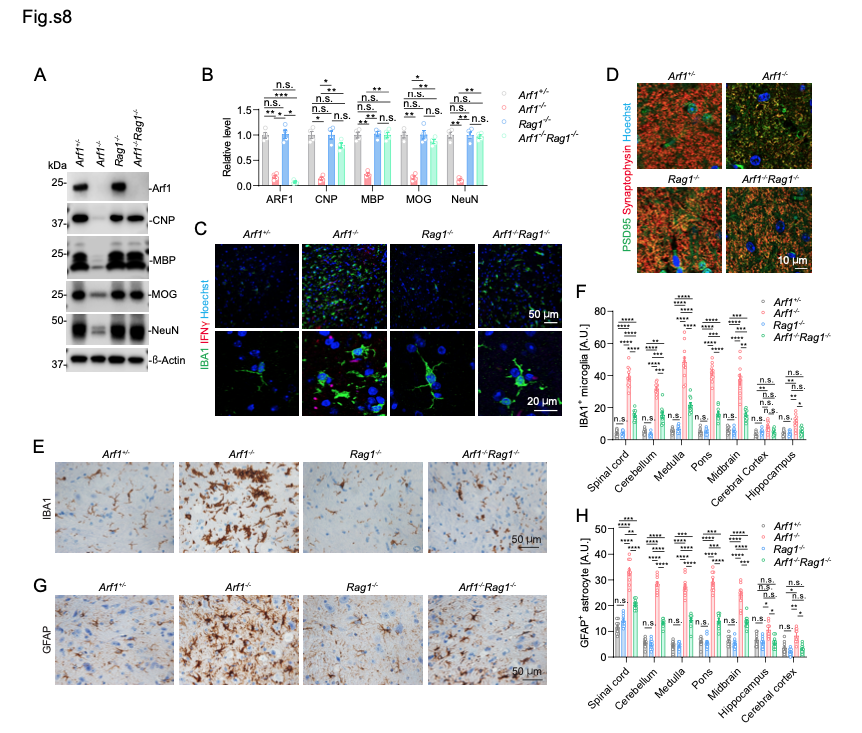


**Supplementary Fig. S8 Rag1 deficiency significantly suppressed the major neurodegenerative phenotypes of ARF1-ablated mice but did not affect ARF1-ablation-induced lipid peroxidation.**

(A, B) Western blotting with indicated antibodies of the spinal cord lysates from mice with indicated genotypes (A). Quantificaiton of western blot (B) (n = 4 per group).

(C) Rag1 deficiency significantly suppressed the increased levels of IFN-γ protein and activated microglia of ARF1-ablated mice. Scale bar: 50 μm (upper), 20 μm (bottom).

(D) Immunofluorescence staining for PSD95 and synaptophysin of spinal cords of mice with the indicated genotypes. Scale bar: 10 μm.

(E) Representative spinal cord sections from mice with the indicated genotypes were analyzed by immunohistochemistry for IBA1 (microglia). Scale bar: 50 μm.

(F) Quantification of IBA1^+^ microglia in different brain areas of mice with the indicated genotypes (n = 10 in each group).

(G) Representative spinal cord sections from mice with the indicated genotypes were analyzed by immunohistochemistry for GFAP (astrocytes).

(H) Quantification of GFAP^+^ astrocytes in different brain areas of mice with the indicated genotypes (n = 10 in each group).

n.s. meas no significant difference, *P<0.05, **P < 0.01, ***P < 0.001, ****P<0.0001 using two-way ANOVA (B,F, and H) with Bonferroni multiple comparison test.

**
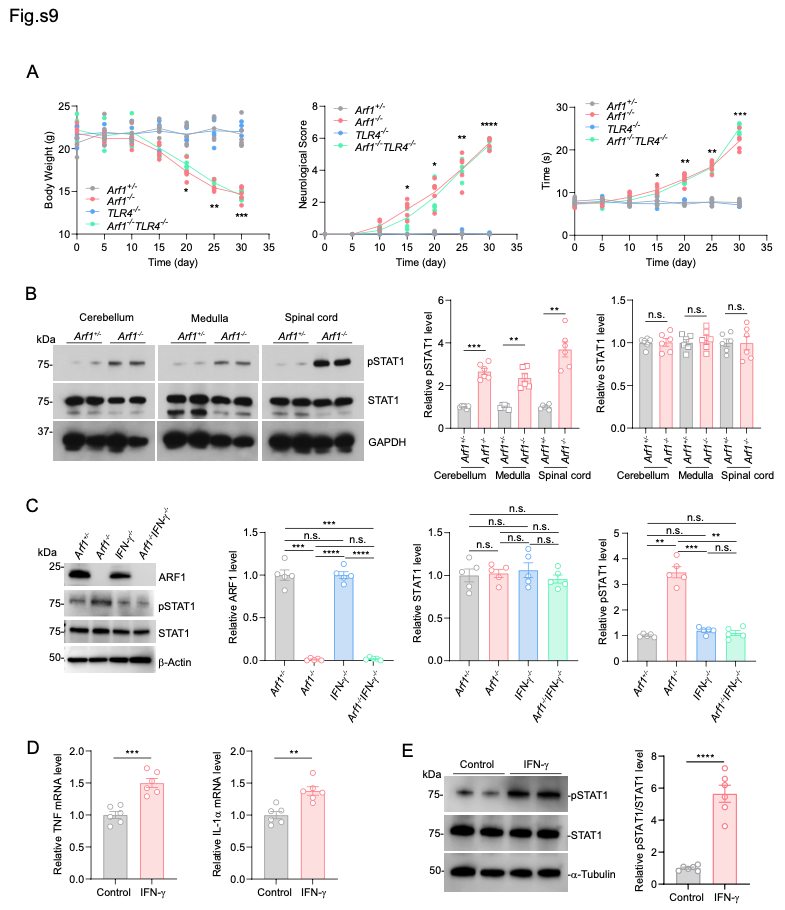
**

**Supplementary Fig. S9 ARF1 ablation induces the reactive astrocyte pathway through the IFNγ–STAT1 pathway.**

(A) Deletion of TLR4 in the ARF1-ablated mice does not rescue the neurodegenerative phenotypes (n = 5 per genotype). Data are represented as mean ± SEM. *P < 0.05, **P < 0.01, ***P < 0.001 using two-way ANOVA with Bonferroni multiple comparison test.

(B) Western blot of phosphorylated STAT1 in lysates of cerebellum, medulla, and spinal cord from mice with the indicated genotypes (n = 6 per group). Data are represented as mean ± SEM. *P < 0.05, **P < 0.01, ***P < 0.001 using two-way ANOVA with Bonferroni multiple comparison test.

(C) The level of phosphorylated STAT1 (p-STAT1) and total STAT1 in indicated mice spinal cord (n = 5 per group). Data are represented as mean ± SEM. n.s. meas no significant, *P < 0.05, **P < 0.01, ***P < 0.001 using one-way ANOVA with Bonferroni multiple comparison test.

(D) TNF and IL-1α in microglia was measured by qRT-PCR (n = 6 per group). Data are represented as mean ± SEM. *P < 0.05, **P < 0.01, ***P < 0.001 using unpaired t-test.

(E) The level of phosphorylated STAT1 (p-STAT1) in control and IFN-γ-treated microglia was analyzed by Western blot analysis, β-Actin served as a loading control (n = 6 per group). Data are represented as mean ± SEM. *P < 0.05, **P < 0.01, ***P < 0.001 using unpaired t-test.


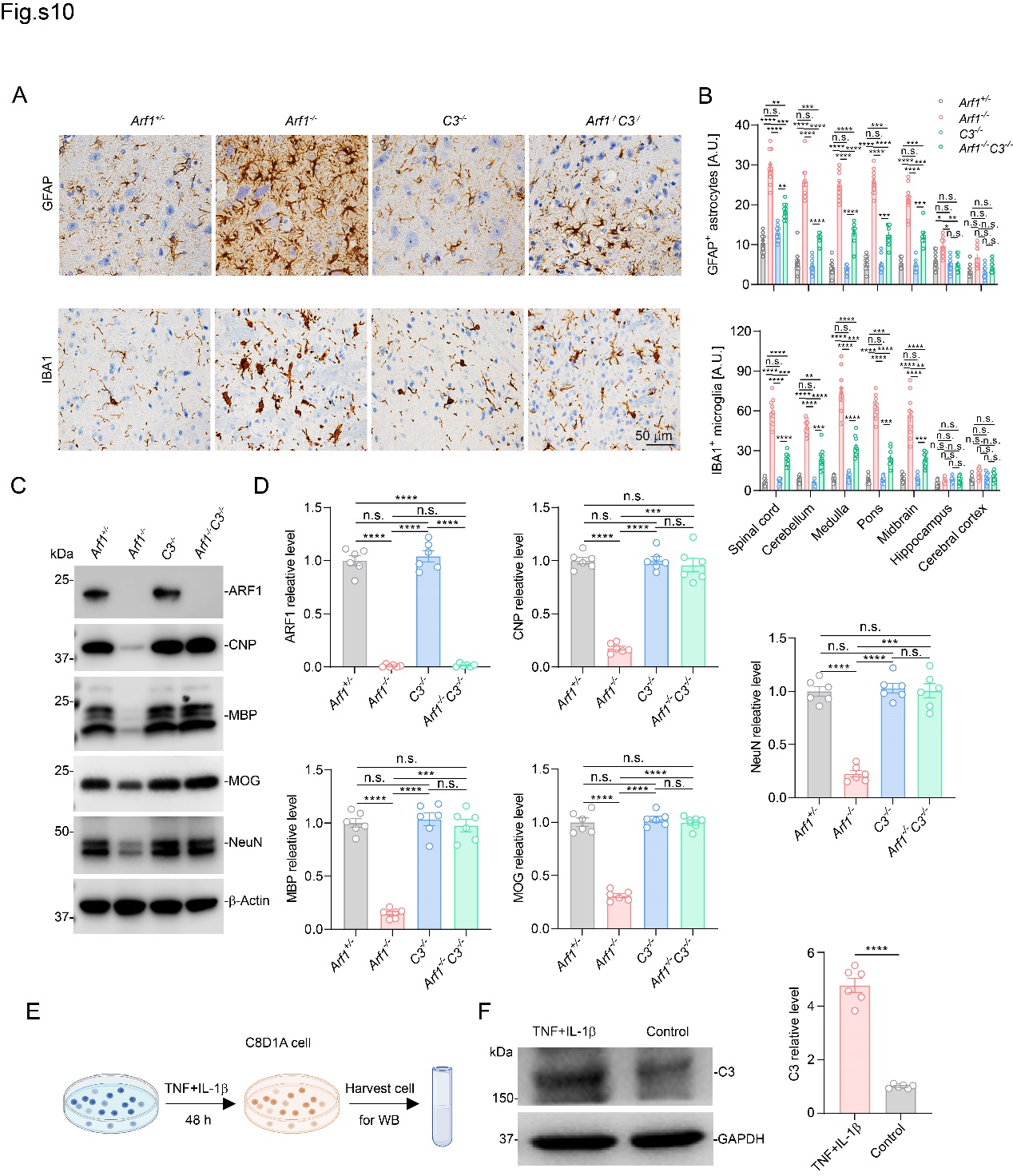


**Supplementary Fig. S10 C3 knockout inhibits the neurodegenerative phenotypes of Arf1-ablated mice.**

(A) Representative spinal cord sections from mice with the indicated genotypes were analyzed by immunohistochemistry for IBA1 (microglia) and GFAP (astrocytes). Scale bar: 50 μm.

(B) Quantification of fig.A. n = 10, Data are represented as mean ± SEM. n.s. meas no significant, *P < 0.05, **P < 0.01, ***P < 0.001, ****P<0.0001 using two-way ANOVA with Bonferroni multiple comparison test.

(C) Western blotting with indicated antibodies of the spinal cord lysates from mice with indicated genotypes.

(D) Quantificaiton of western blot in fig.C (n = 6 per group). Data are represented as mean ± SEM. n.s. meas no significant, *P < 0.05, **P < 0.01, ***P < 0.001, ****P<0.0001 using one-way ANOVA with Bonferroni multiple comparison test.

(E) Experiment set up for identification of TNF and IL-1β iduced the microglia expressed C3 level.

(F) Western blot and quantification of C3 level in cultured C8D1A astrocytes (n = 6). ****P<0.0001 using unpaired t-test.


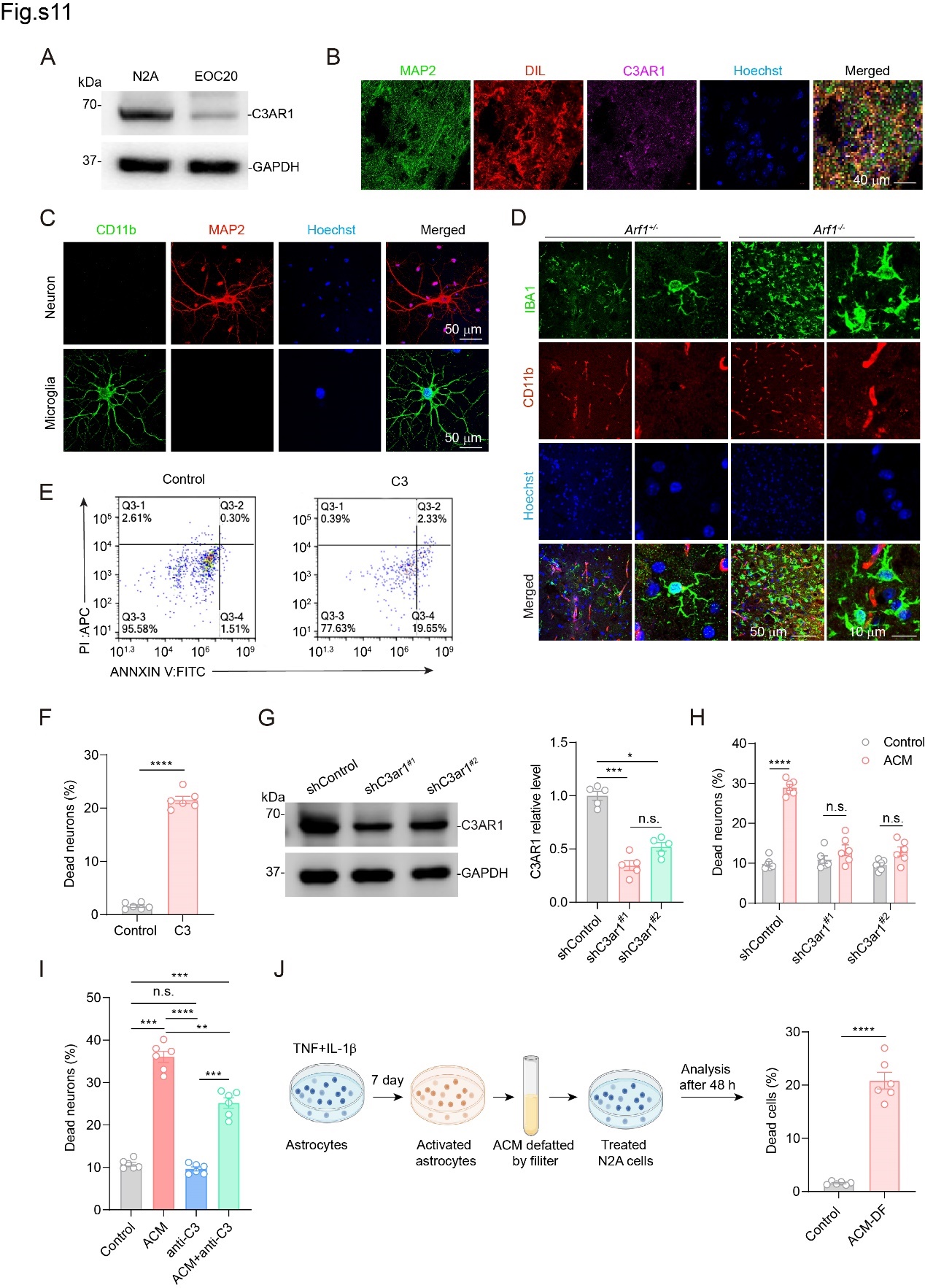


**Supplementary Fig. S11 C3 is the main neurotoxic factor secreted by activated astrocyte.**

(A) Western Blot showed the C3AR1 expressed in N2A and EOC20 cells.

(B) Immunofluorescence stained of MAP2, DIl, C3AR1, and Hoechst in the cerebellum section of mouse.

(C) Immunofluorescence stained of CD11b and MAP2 in the primary cultured neurons and microglia cells. Hoechst stained of nuclear.

(D) Immunofluorescence stained of CD11b and MAP2 in the spinal cord sections from control and *Arf1^-/-^* mice.

(E) Flow cytometry analysis of cultured primary neurons after treatment with purified C3 protein.

(F) Quantitation of ratios of dead neurons on flow cytometry in fig E (n = 6 per group). Data are represented as mean ± SEM. n.s. meas no significant, *P < 0.05, **P < 0.01, ***P < 0.001, ****P<0.0001 using unpaired t-test.

(G) Western blot verified the C3AR1 knockdown efficiency by its shRNAs (n = 5 per group). Data are represented as mean ± SEM. n.s. meas no significant, *P < 0.05, **P < 0.01, ***P < 0.001, ****P<0.0001 using using one-way ANOVA with Bonferroni multiple comparison test.

(H) Quantitation of ratios of dead neurons on flow cytometry. Neurons were transfected with shRNA control (Leucifearse) or C3AR1 shRNAs, and treated with ACM. n = 6. Data are represented as mean ± SEM. n.s. meas no significant, ****P<0.0001 using unpaired t-test.

(I) Quantitation of ratios of dead neurons on flow cytometry from primary cultured neuron at treated with C3 (n = 6 per group). Data are represented as mean ± SEM. n.s. meas no significant, **P < 0.01, ***P < 0.001, ****P<0.0001 using unpaired t-test.

(J) Scheme showed the experimental design of conditional co-culture of astrocyte cells with N2A cells. Quantitation of ratios of dead neurons on flow cytometry (n = 6 per group). ****P < 0.001 using unpaired t-test.

**Supplementary Fig. S12 The ARF1-reduction-induced IFN-γ–reactive astrocyte pathway exists in human diseases.**

(A) Immunofluorescence staining for IFN-γ, IBA1, and Hoechst in pons from normal persons and MS or ALS patients. Scale bar: 50 μm (upper panel), 10 μm (lower panel).

(B) Quantification of IFN-γ-positive dots per image field (40×, n = 12).

(C) Quantification of IBA1-positive microglia per image field (40×, n = 12).

(D) Immunofluorescence staining of synapse and post-synapse with anti-PSD95 and anti-synaptophysin antibodies in the medulla of control, MS, and ALS human specimens. Scale bar: 50 μm (upper panel), 10 μm (lower panel).

(E) Quantification of PSD95-positive and synaptophysin-positive synapses in medulla of control persons, MS patients, and ALS patients (n=10 slides).

(F) Proposed model of ARF1-ablation-induced neurodegeneration.

Data are represented as mean ± SEM. n.s. meas no significant, *P < 0.05, **P < 0.01, ***P < 0.001, ****P<0.0001 using one-way ANOVA with Bonferroni multiple comparison test.

**Supplementary Table** **1** **Information for ablation of ARF1 in various cell types**

| **ID** | **Mice** | **Phenotype** |
| --- | --- | --- |
| JAX:007001 | *UBC-CreER/Arf1^f/f^* | Neurological disorder |
| JAX:012708 | *Thy1-CreER/Arf1^f/f^* | Neurological disorder |
| JAX:003771 | *Nestin-Cre/Arf1^f/f^* | Neurological disorder |
| JAX:005628 | *Emx1-Cre/Arf1^f/f^* | No |
| JAX:003574 | *Alb-Cre/Arf1^f/f^* | No |
| JAX:004586 | *Vilin-Cre/Arf1^f/f^* | A subset of mice exhibit reduced size. |
| JAX:014647 | *Pdx1-Cre/Arf1^f/f^* | No |
| NGI:2664968 | *Foxa3-Cre/Arf1^f/f^* | Mice perished during the embryonic stage |
| JAX:024098 | *GFAP-CreER/Arf1^f/f^* | No |
| JAX:032770 | *Pdgfra-CreER/Arf1^f/f^* | No |
| JAX:005975 | *Plp1-CreER/Arf1^f/f^* | No |
| JAX:027651 | *Sox10-CreER/Arf1^f/f^* | No |
| JAX:031674 | *LysM-CreER/Arf1^f/f^* | No |
| JAX:021160 | *CX3CR1-CreER/Arf1^f/f^* | No |
| JAX:031820 | *TMEM119-CreER/Arf1^f/f^* | No |
| JAX:036499 | *Aire-CreER/Arf1^f/f^* | No |
| JAX:003802 | *Lck-Cre/Arf1^f/f^* | No |

Note: The "ID" refers to the identification number assigned to Cre or CreER mice at The Jackson Lab, while "NGI" pertains to the NIH/NCI mice ID number. Cre mice, bred with Arf1f/f, were observed for over five months, displaying no discernible phenotype and thus categorized as having no phenotype. On the other hand, CreER mice, crossed with Arf1f/f, underwent tamoxifen injection over a span of five days. Subsequently, these mice were closely monitored for an additional 3-5 months.

**Supplementary Table 2 Information for Human Specimens**

| **SID** | **GUID** | **BW** | **DISORD**  **ER** | **AGE**  **YEARs** | **AGE**  **DAYs** | **SEX** | **RACE** | **HIV** | **HBsAG** | **PMINT**  **ERVAL** |
| --- | --- | --- | --- | --- | --- | --- | --- | --- | --- | --- |
| 1491 | NDAR_INVBP614GMR | N/A | MS | 55 | 14 | Male | White | N/A | N/A | 3 |
| 5274 | NDAR_INVTZ491ZPW | N/A | Control | 64 | 187 | Female | White | Negative | Negative | 20 |
| 5487 | NDAR_INVZZ586AF4 | 919 | MS | 71 | 124 | Female | Black | N/A | N/A | 44 |
| 5781 | NDAR_INVDB589AU8 | 1342 | ALS | 61 | 201 | Female | White | N/A | N/A | 3 |
| 5828 | NDAR_INVUL149RBY | 1350 | Control | 66 | 237 | Female | White | N/A | N/A | 25 |
| 5846 | NDAR_INVNR516VGD | 765 | MS | 63 | 167 | Female | White | N/A | N/A | 6 |
| 5898 | NDAR_INVAA510YXW | 1136 | ALS | 68 | 282 | Female | Asian | Negative | Negative | 17 |
| 6065 | NDAR_INVBK320LJG | 1320 | Control | 55 | 181 | Male | White | Negative | Negative | 22 |
| 6195 | NDAR_INVEBAKXX7M | 1302 | ALS | 55 | 10 | Male | Unknown | N/A | N/A | 24 |

Note:
SID: Subject Identification number; GUID: Globally Unique Identifier; BW: Brain Weight(g); HIV: Human immunodeficiency virus; HBsAG: Hepatitis B surface antigen; N/A: not available; MS: Multiple Sclerosis; ALS: Amyotrophic Lateral Sclerosis; Control: Unaffected Control.

**REFERENCES**

41. Manglani M, Gossa S, McGavern DB. Leukocyte Isolation from Brain, Spinal Cord, and Meninges for Flow Cytometric Analysis. *Curr Protoc Immunol* 2018; **121**: e44. 10.1002/cpim.44
